# Assessing the temporal clustering of coastal storm tide hazards under natural variability in a near 500-year model run

**DOI:** 10.1007/s10236-025-01766-4

**Published:** 2026-02-09

**Authors:** Luke J. Jenkins, Ivan D. Haigh, Hachem Kassem, Douglas Pender, Jenny Sansom, Rob Lamb, Tom Howard

**Affiliations:** 1 School of Ocean and Earth Science, University of Southampton, National Oceanography Centre, European Way, SO14 3ZH Southampton, UK; 2 JBA Consulting, Unit 2.1, Quantum Court, Heriot Watt Research Park, EH14 4AP Edinburgh, UK; 3 Environment Agency, Lateral House, 8 City Walk, Leeds, LS11 9AT UK; 4 JBA Trust, 1 Broughton Park, Skipton, BD23 3FD UK; 5 Lancaster Environment Centre, Lancaster University, Lancaster, LA1 4YQ UK; 6 Met Office Hadley Centre, FitzRoy Road, Exeter, EX1 3PB UK

**Keywords:** Storm clustering, Natural variability, Ocean modelling, Coastal hazards, Coastal management

## Abstract

The temporal clustering of storms can present successive natural hazards for coastal areas in the form of extreme sea levels, storm surges and waves. Studies have investigated the prevalence of the temporal clustering of such hazards but are hindered by the rarity of the phenomena combined with short records and a lack of data availability around the coastline. This has made it difficult to determine if the levels of clustering reported were typical for the location or were being masked by natural variability or climate change over different timescales. In this study, we assess a near 500-year model simulation of extreme sea levels and storm surges forced with pre-industrial meteorological conditions to quantify the levels of temporal clustering seen from natural variability around Great Britain. We then utilise a 50-year rolling window to see how clustering statistics can change through time when dealing with time periods that are representative of the average length of a record in the United Kingdom National Tide Gauge Network. When using near 500-year timeseries, we highlight that many clustering statistics return values close to their statistical expectancies. However, when analysing discrete 50-year windows, results can vary dramatically. The percentage of years with an extreme sea level or surge exceedance at a given location at the 1 in 1-, 5-, and 10-year return level, can vary by up to ~ 33%, ~ 24%, and ~ 18%, the mean number of days between consecutive sea level or surge exceedances can vary by ~ 231, ~14,780, and ~ 17,793 days, and the extremal index can vary by ~ 0.37, ~ 0.64, and ~ 0.79, respectively. Although these results represent the best estimate of the levels of clustering to be expected under natural variability, a comparison of the longest records in the tide gauge network and their nearest model grid nodes shows a tendency for the model to underestimate the clustering statistics that are calculated from the measured data (apart from the extremal index). As such, these can be considered to represent the minimum levels of temporal clustering around Great Britain, as the potential underestimation of clustering, combined with climatic change and sea level rise, means that the temporal clustering of sea levels and storm surges are likely to be far greater over the next 500 years.

**Supplementary Information:**

The online version contains supplementary material available at 10.1007/s10236-025-01766-4.

## Introduction

Coastal regions of the world are of vital social and economic importance. They are home to a disproportionate amount of the world’s population with more than 50% of coastal countries having over 80% of their population within 100 km of the coastline, with many people living in coastal cities (Martínez et al. 2007). These cities form important economic centres, with marine economic processes stretching as far as 150 km inland (Mikhaylov and Plotnikova 2021). They have experienced significant population growth that is predicted to continue to increase into the future (Maul and Duedall 2019). There are numerous natural hazards that coastal regions face (e.g., coastal flooding, erosion, salt water intrusion), being some of the most high-risk regions of the world (Kron 2013), but the projected population development presents a significant increase in risk to future coastal populations as the climate changes and sea levels rise (Neumann et al. 2015; Reimann et al. 2023).

The primary natural hazard for coastal populations is flooding. Over the last century, the average annual number of people affected by storm-tide driven flood events was 1.5 million (Bouwer and Jonkman 2018), and recently there has been an increase in the mortality fraction per event for low-income countries (Jonkman et al. 2024). Kirezci et al. (2023) estimate that the number of people affected globally by coastal flooding in 2015 was 34 million and predict that, by 2100, this could rise to 246 million people if no further adaptation was taken. Nearly half of global assets (when defined as 2.8 multiplied by the population multiplied by the gross domestic product) could be at risk in 2100 with the majority of coastal flooding being caused by storm-tide events (Kirezci et al. 2020).

The impacts of flooding in coastal regions can be amplified when two or more flood events occur in a given locality in quick succession in time (i.e., a few days apart). The temporal clustering of sea level, storm surge and wave hazards can undo natural and anthropogenic recovery and repair time, leaving coastal communities vulnerable to the repeat floods. Storm clustering has been observed in Northwest Europe, when multiple extratropical storms propagate over the region in succession – the chief example being the 2013/14 winter season, where, on average, 1 intense storm impacted the UK coast every 2.5 days between December and February, and the UK saw its stormiest winter season on record (Matthews et al. 2014; Priestley et al. 2017). The flooding in this season caused economic damages of around £1,300 billion for the UK, with 25% of total damages suffered by 10,465 residential properties (Chatterton et al. 2016). Storm clustering over the North Atlantic has been linked to specific atmospheric patterns and ocean-atmosphere teleconnections, and although there is uncertainty in regard to changes to the prevalence of storm clustering with climate change (Dacre and Pinto 2020), the 2013/14 winter season has been shown to have been more likely by human anthropogenic emissions relative to a pre-industrial climate (Schaller et al. 2016; Kay et al. 2018). With rising sea levels, the storm surge input required to take sea levels over critical thresholds will reduce, so the levels of temporal clustering seen in coastal flooding will undoubtedly increase in frequency in the future.

Previous research has analysed the temporal clustering of coastal hazards that cause flooding, focusing on sea levels, storm surges, and waves. For example, clustering of coastal hazards in New Zealand have been related to ocean-atmosphere teleconnections and mean sea level anomalies, with an increase in clustered storms in the Southwest during El Niño, and in the North during La Niña (Godoi et al. 2018; Stephens et al. 2020). In the Mediterranean, Besio et al. (2017) used Allan variance to measure the temporal clustering of waves, finding seasonality over the whole basin and clustering over smaller timescales in more localized areas. Extreme sea levels in the Adriatic Sea have been shown to occur simultaneously on large spatial scales and to cluster over very short timescales of a few days (Sepic et al. 2022). Much of the research into the temporal clustering of coastal hazards has been centred on the UK, particularly after the extreme 2013/14 winter season brought significant attention to the topic. The clustering of sea levels and storm surges were analysed by Wadey et al. (2014) and Haigh et al. (2016), the latter of whom determined that the vast majority of high sea levels were determined by high tides combining with surges rather than extreme surges alone. They showed that the quick-succession clustering of sea levels was therefore prevented from occurring within 4–8 days due to the spring-neap tidal cycle. The clustering of waves have been analysed by Malagon Santos et al. (2017) and Dhoop and Mason (2018), who showed that wave extremes, unlike sea levels, had more similar event return times across sites and were seen to cluster on timescales within the spring-neap tidal cycle. Recently, Jenkins et al. (2023) examined the clustering of sea levels, storm surges, and waves using a consistent approach across the UK’s entire observational record and found that all three parameters clustered on interannual and intra-annual timescales across the whole of the UK.

With rising sea levels and potential changes in storminess, it is likely that temporal clustering seen in extreme sea levels and storm surges will increase in the future. A key unresolved question, is how much clustering should we expect to see irrespective of this? The previous research into the clustering of coastal hazards in the UK, summarised above, quantified the interannual and intra-annual clustering that has been seen, with the 2013-14 winter season clearly standing out. This winter season was unique in the observational record in its storminess, with unprecedented cyclone intensity and frequency (Matthews et al. 2014) that saw the highest number of sea level, surge, and wave height extremes (Jenkins et al. 2023). However, with an average tide gauge record length of ~ 50 years in the UK National Tide Gauge Network (UKNTGN), it is difficult to frame the season as either a one-off extreme event or something that should be expected. The combination of such short records, missing data, different data lengths, and a lack of spatial coverage, means that observational studies cannot fully capture the picture of the clustering of coastal hazards.

Therefore, our overall aim in this paper is to characterise the levels of temporal clustering of extreme sea levels and storm surges in Great Britain under natural variability. We expand upon the work of Jenkins et al. (2023), whose analysis focused on the short observational record and a 40-year hindcast, by utilising a model simulation of sea level and storm surge under pre-industrial conditions, providing nearly 500-years of synthetic data at a high spatial resolution around the coastline of Great Britain, without the influence of mean sea level rise or climate change. We have three objectives, as follows:

1. To quantify the levels of interannual and intra-annual clustering of sea levels and non-tidal residuals under natural variability;
2. To analyse how clustering statistics vary through time depending on the time period chosen; and
3. To compare the levels of clustering seen in the model data to the longest tide gauge records in the UKNTGN.

One issue posed by Jenkins et al. (2023), when analysing the clustering of extreme sea levels and surges around the UK, was the lack of historical context that observed periods of clustering could be framed within, due to limited observational record lengths and data gaps. To address this, here, we apply a 50-year discrete rolling window to our clustering statistic calculations and evaluate the range of results that have been gained from the 435 50-year time periods around Great Britain. This enables us to highlight the potential difficulties of understanding clustering on the relatively small timescales of observed data records. From this, assumptions can be made on how to generally characterise an observed record’s level of clustering of extremes. Consequently, we compare the levels of clustering calculated from rolling ~ 50-year windows to results from the same rolling window analysis of the longest observational records in the UK. These findings quantify the expected occurrence of clustering of coastal hazards around the UK, helping to inform coastal engineers, managers, and planners as they evaluate the threat of storm surges and extreme sea levels clustering over varying timescales.

## Data

This study uses two main datasets, sea levels and tides from a modelling hindcast, and measured sea levels from tide gauge records. Each dataset is described in turn below.

### Modelled datasets

To analyse the clustering of coastal storm-tide hazards over a large timescale, we utilise the simulated model storm surge dataset from Howard and Williams (2021). The authors used a process-based numerical climate model to force a coastal shelf sea model and create a near 500-year (483) dataset of synthesised storm surges for the entire Great Britain region. The influence of anthropogenic climate change is not included in the climate model, and no change in mean sea level was present in the shelf sea model. Therefore, the dataset provides a unique opportunity to analyse the behaviour of storm-tide hazards that are driven purely by natural variability. The models and data output format are now briefly discussed; for full details see Howard and Williams (2021).

To force the shelf sea model and generate the storm surge dataset, Howard and Williams (2021) used a 483-year control simulation from the Hadley Centre Global Environment Model in the Global Coupled configuration 3, medium resolution atmosphere, medium resolution ocean (HadGEM3-GC3-MM) numerical climate model (Williams et al. 2018) with fixed pre-industrial greenhouse gas concentrations. The atmospheric horizontal resolution of this model is ~ 60 km in the mid-latitudes and the ocean horizontal resolution is around $\:1/4$°. Williams et al.‘s (2015) paper illustrates that the HadGEM3-GC3-MM had greater success in accurately representing North Atlantic storm tracks compared to its predecessors, with increased synoptic variability, and Howard and Williams (2021) conclude that this suggests surface wind and pressure forcing by HadGEM3-GC3-MM could produce realistic UK storm surge simulations. They also note that a global climate model may be considered too coarse in its spatial resolution for the purpose of assessing UK storm surges and address this by pointing out that: (a) storm surges in the UK are usually driven by large-scale processes that are well-represented in atmospheric models; (b) a storm surge integrates winds and pressures over large spatial and temporal scales, meaning that smaller-scale processes are relatively less important; and (c) the issues with orographic drag have negligible effect on the open sea where storm surges are typically generated (Howard and Williams 2021).

The climate forcings of HadGEM3-GC3-MM were applied to the National Oceanography Centre’s Continental Shelf 3 (CS3) model (Flather et al. 1991), a depth-averaged barotropic coastal shelf sea model that has an external surge component and an open boundary input of the largest 15 tidal harmonics, to simulate storm tides around the UK. CS3 represents one of the most well-validated operational storm surge forecasting models and was used by the Met Office in the UK operationally for ~ 7 years (Horsburgh et al. 2021). CS3 covers the European shelf region from 48 to 63 N and 12 W-13E, with a horizontal resolution of around $\:1/9$° latitude by $\:1/6$° longitude (~ 12 km). The accuracy of the total water levels that the model produces varies around the coast but typically sees root mean square errors in the order of 10 cm when forced with numerical atmospheric data (Flather 2000).

**Fig. 1:**
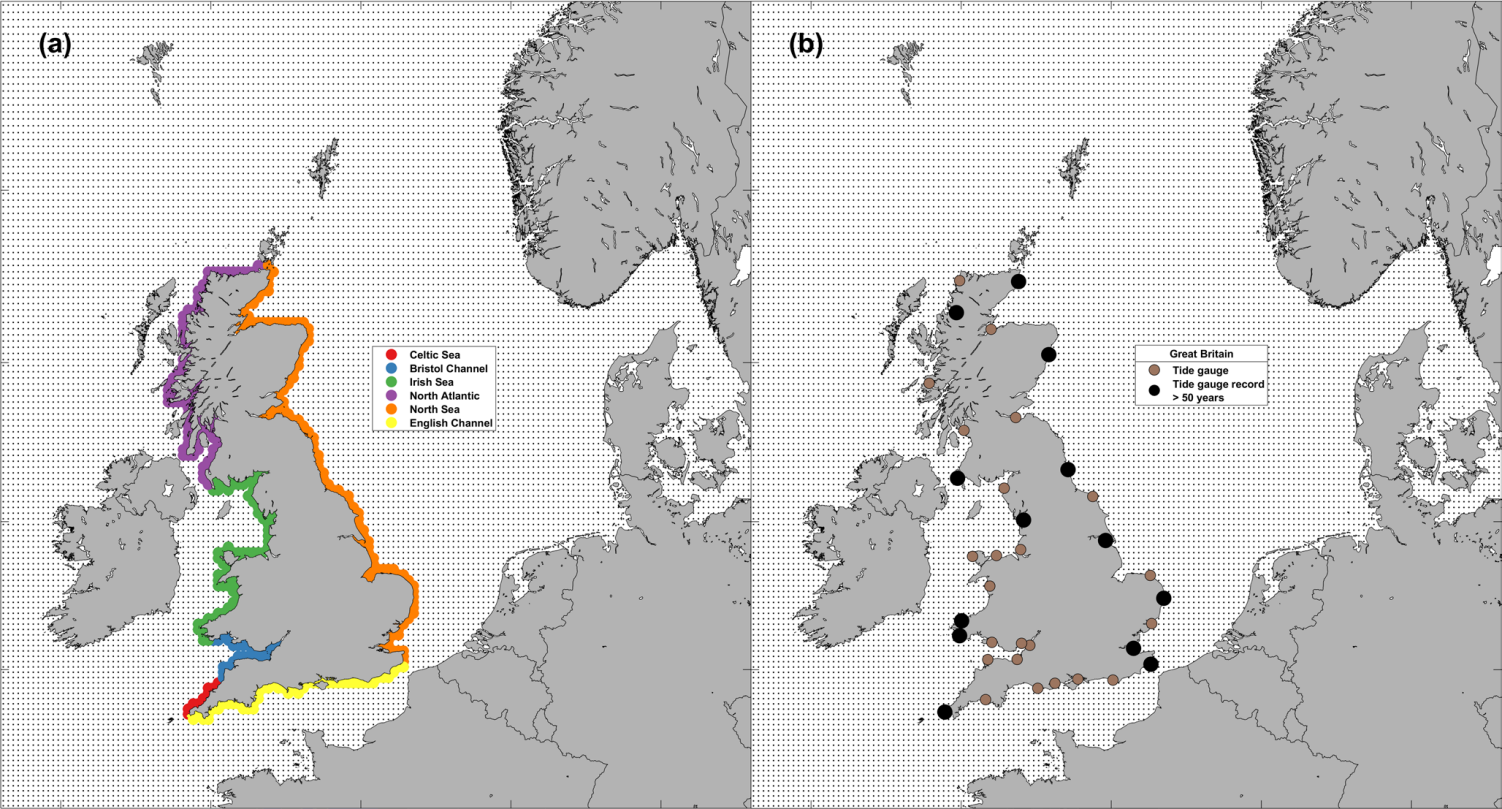
**a** Model grid nodes for the Great British coastline used in this study highlighted by region (**b**) UKNTGN tide gauges on the Great British coast, and the records over 50 years in length used in this study. The background dots in both panels represent all the model grid nodes

Two model runs were undertaken, one with tidal forcing only and one with both tidal and meteorological forcings. The output data from the two model runs consisted of 135 × 150 latitude and longitude grids, with each grid node containing a timeseries of either tide, or tide and surge (i.e., sea level), on an hourly temporal resolution for a period of 483 years (1851–2334). The model domain and grid nodes are shown in Fig. 1, with the Great Britain coastal grid nodes used in this research highlighted in Fig. 1a, whilst Fig. 1b shows the location of UKNTGN tide gauge sites in Great Britain, with those with records of > 50 years used in this research also highlighted.

### Measured datasets

Regarding the measured datasets of sea levels, we use records from the UKNTGN. The British Oceanographic Data Centre (BODC) are responsible for the data retrieval, monitoring, and quality control of the tide gauge network and we obtained the data from their website (www.bodc.ac.uk). The network consists of 47 gauges, 3 of which only contain historical data, but the rest are operational and monitored and maintained. The tide gauges have an hourly temporal resolution prior to 1993, and a 15-minute temporal resolution thereafter. Data are provided up to November 2023. Data flagged by the BODC as being suspect were removed prior to the analysis. We chose the longest records in the UKNTGN for Great Britain to compare against the modelled data in our 50-year rolling window analysis. These sites were: Milford Haven, Fishguard, Heysham, Portpatrick, Ullapool, Wick, Aberdeen, North Shields, Immingham, Lowestoft, Sheerness, Dover, and Newlyn. The locations of the Great Britain coastal tide gauges used in this study are presented in Fig. 1b, with the durations, data coverages, and number of rolling windows of each tide gauge are shown in Table 1.

**Table 1:** Longest sea level data records in the UKNTGN used in this research

Site	Length of record (years)	Number of 50-year rolling window periods	Data coverage (%)
Aberdeen	78	28	74.24
Dover	74	24	74.88
Fishguard	60	10	80.74
Heysham	59	9	78.35
Immingham	65	15	81.16
Lowestoft	60	10	95.62
Milford Haven	63	13	79.65
Newlyn	109	59	88.48
North Shields	70	20	79.24
Portpatrick	56	6	86.04
Sheerness	57	7	63.56
Ullapool	55	5	80.76
Wick	58	8	92.11

## Methods

We initially follow an analysis approach similar to Jenkins et al. (2023), with some amendments, before undertaking more detailed analysis tailored to the characteristics of the modelled dataset. The data preparation is described in Sect. 3.1. The statistical approaches we used to quantify clustering are outlined in Sect. 3.2., as well as the approach we used to examine changes through time, via a rolling window.

### Data preparation

The two sea level-derived parameters of most interest to storm-tide hazard research are the non-tidal residual and the still sea level. Changes in sea surface height without the influence of wind waves are referred to as the still sea level, whereas the non-tidal residual is the difference between the tidal level and the still sea level. The non-tidal residual contains the component of sea level driven primarily by meteorology (i.e., the storm surge or simply surge), but may contain errors resulting from harmonic prediction, timing, or non-linear interactions (Horsburgh and Wilson 2007). As surge is chiefly driven by meteorology, it is greatly affected by storm clustering. Still sea level, on the other hand, is also primarily driven by astronomical forces (which are typically larger than the non-tidal residual around the majority of the UK coast) and thus is influenced less by meteorology. Still sea level is the totality of what is driving coastal flooding, so it is imperative to analyse its temporal clustering. For the modelled data, the timeseries at each model grid node contain both still sea level and the tidal components. To get the surge component, we simply subtract the tides from the still sea levels leaving the non-tidal residuals. For the measured data, we first remove mean sea level (MSL) trends from the timeseries (the modelled data does not have MSL trends present). We remove MSL trends using the same method as Jenkins et al. (2023), calculating annual trends using linear regression at each tide gauge, interpolating these onto hourly timeseries, and then removing them. Hereafter, ‘sea level(s)’ refer(s) to still sea levels for the modelled data or still sea levels with MSL trends removed for the measured data. Next, to get the measured surge component, we generate timeseries of reconstructed tide for each tide gauge that we can then subtract from the sea level timeseries to leave the non-tidal residual. Using the MATLAB Unified Tidal Analysis and Predictions Functions (Codiga 2022), for each tide gauge we do a separate harmonic analysis and tidal reconstruction for each calendar year, with the nearest year that meets the data coverage checks (≥ 75% of data) instead being used for the harmonic analysis if the target year does not meet those checks. Subtracting the tide timeseries from the measured sea level provided the surge component, namely the non-tidal residual.

**Fig. 2:**
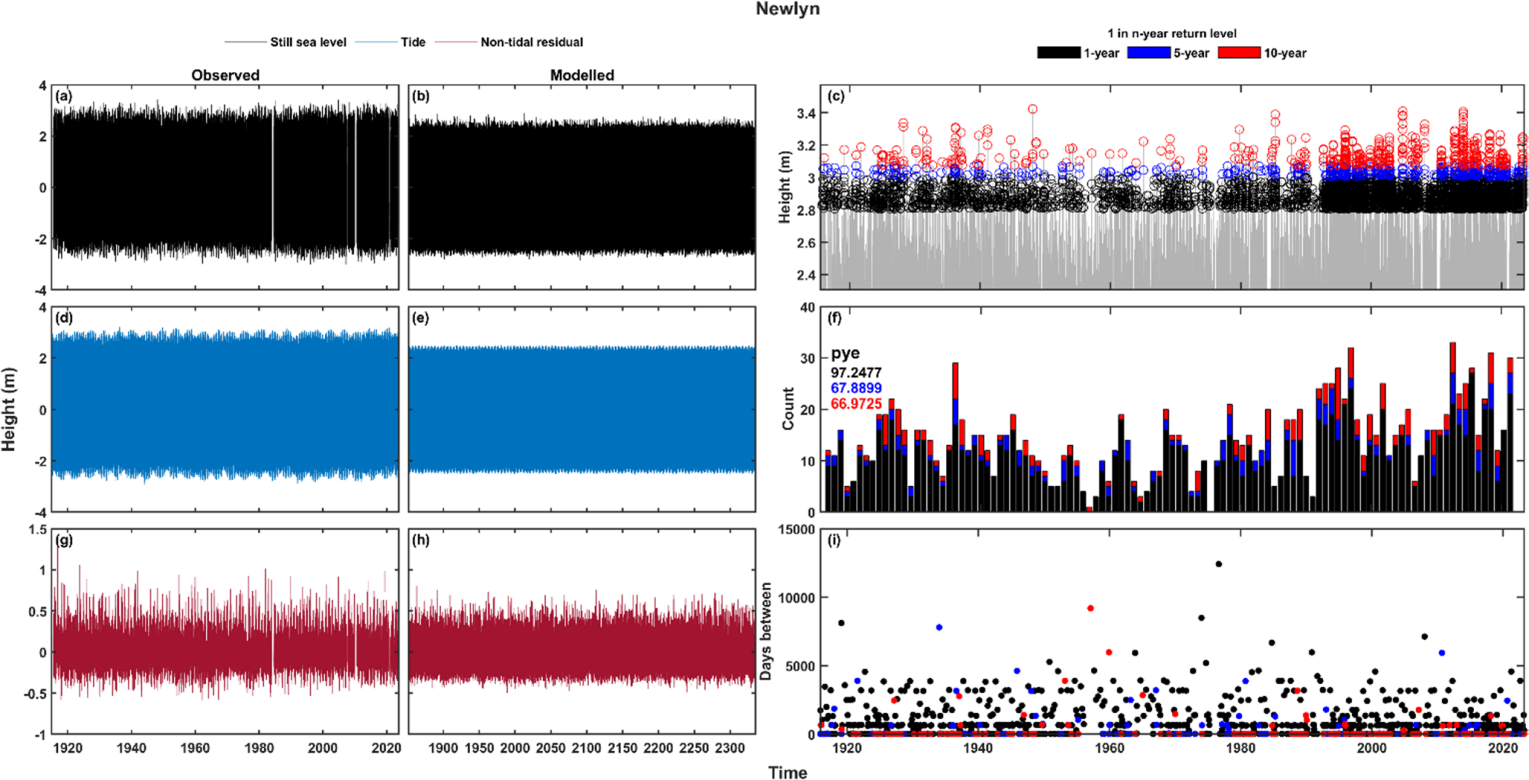
Timeseries example of observed (**a**, **d**, and **g**) and modelled (**b**, **e**, and **h**) data at Newlyn. (**a**-**b**) still sea level, (**d**-**e**) tide, and (**g**-**h**) non-tidal residual. Exceedance data for the observed sea level data at the three return level thresholds is shown in the right column, with declustered exceedances (**c**), number of events per storm year (with the percentage of storm years with an exceedance, pye, shown) (**f**), and days between consecutive exceedances (**i**)

To identify extreme still sea level and non-tidal residual events, we first calculate exceedance probabilities (return period levels) for every grid point. We include the 1 in 1- and 1 in 5-year return level thresholds that were used in Jenkins et al. (2023) as the levels of clustering seen in their research were greater at lower event magnitudes. However, due to the temporal length of the data used here, we also consider the higher magnitude threshold of the 1 in 10-year return level. To calculate the return levels, we follow the peaks over threshold (POT) and storm window approach of Haigh et al. (2016) and Jenkins et al. (2023). First, the timeseries is sorted to be descending in magnitude, then the timeseries is worked through in this order, removing all values within a *n*-hour storm window of the target value, leaving the declustered values. A storm window of 32 h was used as this has been seen to best represent UK extreme sea levels and storm surges (Jenkins et al. 2023). Next, a threshold is set to subsample the declustered data, in this study we choose the percentile (from the initial timeseries) that gives an average of 3 declustered exceedances per storm year (see below). Finally, a generalised Pareto distribution (GPD) is fit to the subsampled POT values to give estimates of return level thresholds that use the last year in the timeseries as the base year. An example timeseries of the measured and modelled data at Newlyn is shown in Fig. 2, with still sea levels (Fig. 2a-b), tide (Fig. 2d-e), and surge (non-tidal residual) (Fig. 2g-h) shown, as well as measured sea level exceedances (Fig. 2c), percentage of years with an exceedance (Fig. 2f), and days between consecutive exceedances (Fig. 2i).

### Clustering statistics and changes through time – rolling window

We use three statistical approaches to quantify levels of clustering. Each of these methods is described in turn in the sub-sections below. For the rolling window analysis, the modelled data were split into 435 blocks of 50-years each, with the same methodology described in this section applied to each block in turn to gain return levels and clustering statistics. This 50-year rolling window was chosen as this is close to the average length of the UKNTGN tide gauge records and can therefore best illustrate the suitability of such record lengths to determine clustering.

#### Exceedance counts (percentage of years with an exceedance)

First, we analyse the levels of clustering seen on interannual timescales. Jenkins et al. (2023), we also utilise the ‘storm year’ approach, where annual periods are defined as being from the 1 st of July to the 30th of June the next year, rather than a calendar year. This enables each annual period to encompass the North Atlantic winter storm season, which is when most storms occur and is thus an appropriate grouping period for clustering research. Note, we also take this approach for all given time periods that are usually defined by the Gregorian calendar. For example, the 50-year rolling windows start from the 1 st of July of the first year and ended on the 30th of June of the fiftieth year. We calculate the percentage of years with an exceedance to highlight the levels of interannual clustering seen throughout the timeseries. If clustering was present, one would expect a lower percentage of years with an exceedance value as that would demonstrate that the exceedances were not as uniformly distributed throughout the timeseries, instead they were clustered within storm years.

#### Days between consecutive exceedances

Second, we count the number of days between consecutive exceedances, continuing to use descriptive statistics to analyse the levels of clustering seen on intra-annual timescales. Again, intra-annual in this research refers to events within a storm year (not a calendar year). We simply count the number of days between consecutive exceedances, calculating the mean.

#### Extremal index

Third, we analyse the clustering of extreme values by computing the extremal index ($\:\theta\:$), which measures the degree of clustering of extremes of a stationary process. Here we utilise the R *exdex* package (Northrop and Christodoulides 2023), which makes frequentist inferences of the extremal index. We focus on the provided threshold-based extremal index methodologies that analyse the distribution of inter-exceedance times of observations above a threshold ($\:u$) (Ferro and Segers 2003). Specifically, we use the ‘K-gaps’ method of Süveges and Davison (2010) which uses a run parameter ($\:K$) that declusters exceedances by setting all inter-exceedance times that are $\:\le\:K$ to zero. By setting the $\:u$ and $\:K$ values to our return levels and storm window, respectively, we can gain a statistical measure of how often our declustered exceedances, that represent individual storms, cluster throughout an entire timeseries. We also employ the similar ‘D-gaps’ method of Holešovský and Fusek (2020), where the $\:K$ parameter is instead replaced by the artificial censoring parameter $\:D$, which instead of inter-exceedance times $\:\le\:D$ being considered zero, these times are considered left-censored and contribute to the log-likelihood only by providing information that they are $\:\le\:D$. We again set the *u* and *D* values to our return levels and storm window, respectively. For both ‘K-gaps’ and ‘D-gaps’, *exdex* provides maximum likelihood estimates of the extremal index ($\:\theta\:$). There was no distinguishable difference in the results of the analyses that used K-gaps and D-gaps, so we therefore present the D-gaps results in the paper with the K-gaps results available in the supplementary materials.

## Results

We present the results for each of the clustering statistics (percentage of storm years with an exceedance, mean number of days between consecutive exceedances, and the extremal index) in turn in Sect. 4.1. First, discoursing the results for non-tidal residual and sea level for the whole timeseries for the parameter, then discoursing the values seen when applying the rolling window. Figures in this section show results that have been calculated for the whole timeseries in colour whereas results derived from the rolling window analysis are presented in grey. Next, in Sect. 4.2, we again consider each clustering statistic in turn, comparing the differences in the rolling window values between the measured and modelled data.

### Levels of interannual and intra-annual clustering

**Fig. 3:**
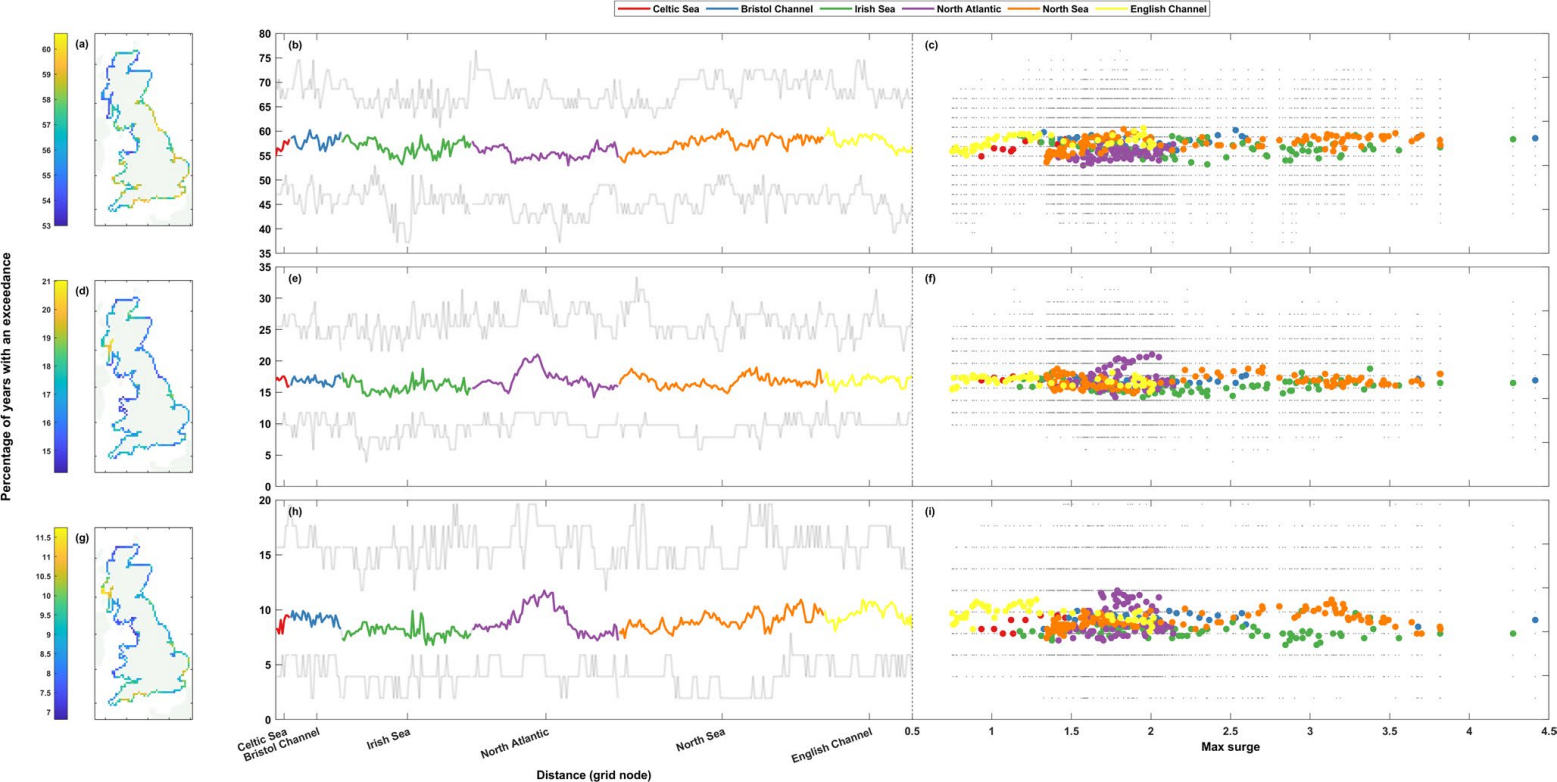
Percentage of storm years with non-tidal residual exceedance. The top row (panels **a**-**c**) shows results at the 1 in 1-year return level, the middle row (**d**-**f**) the 1 in 5-year return level, and the bottom row (**g**-**i**) the 1 in 10-year return level. Percentages are shown spatially in panels **a**, **d**, and **g** (left column), clockwise around the coast from the Southwest tip of England ending back at Newlyn in panels **b**, **e**, and **h** (middle column), and finally shown as a scatter plot against maximum non-tidal residual at that grid node in panels **c**, **f**, and **i** (right column). The grey lines (dots) in panels **b**, **e**, and **h** (**c**, **f**, and **i**) show the range of (all) results from the 50-year rolling window analysis

**Fig. 4:**
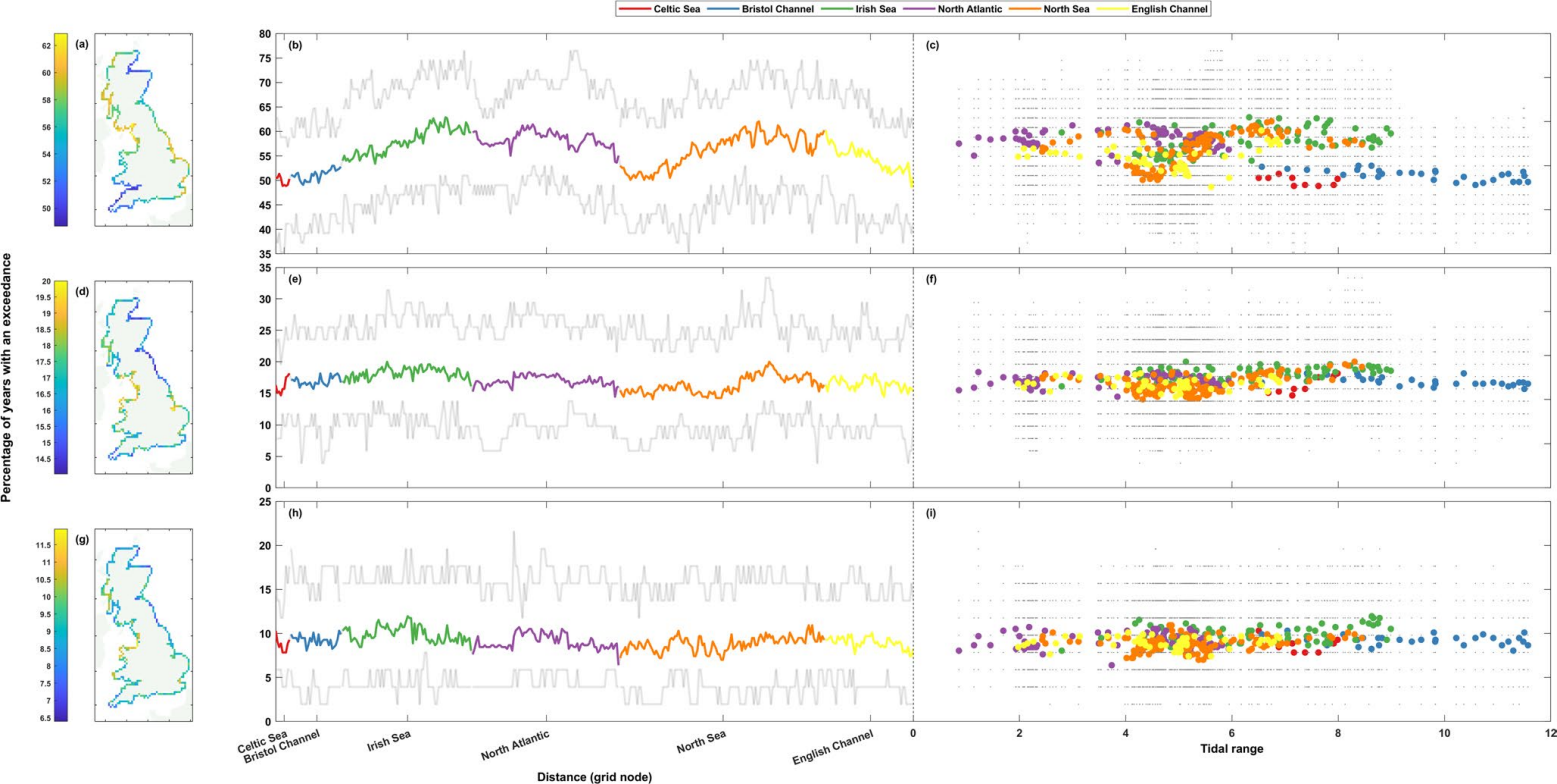
Percentage of storm years with sea level exceedance. The top row (**a**-**c**) shows results at the 1 in 1-year return level, the middle row (**d**-**f**) the 1 in 5-year return level, and the bottom row (**g**-**i**) the 1 in 10-year return level. Percentages are shown spatially in panels **a**, **d**, and **g** (left column), clockwise around the coast from the Southwest tip of England ending back at Newlyn in panels **b**, **e**, and **h** (middle column), and finally shown as a scatter plot against tidal range at that grid node in panels **c**, **f**, and **i** (right column). The grey lines (dots) in panels **b**, **e**, and **h** (**c**, **f**, and **i**) show the range of (all) results from the 50-year rolling window analysis

**Fig. 5:**
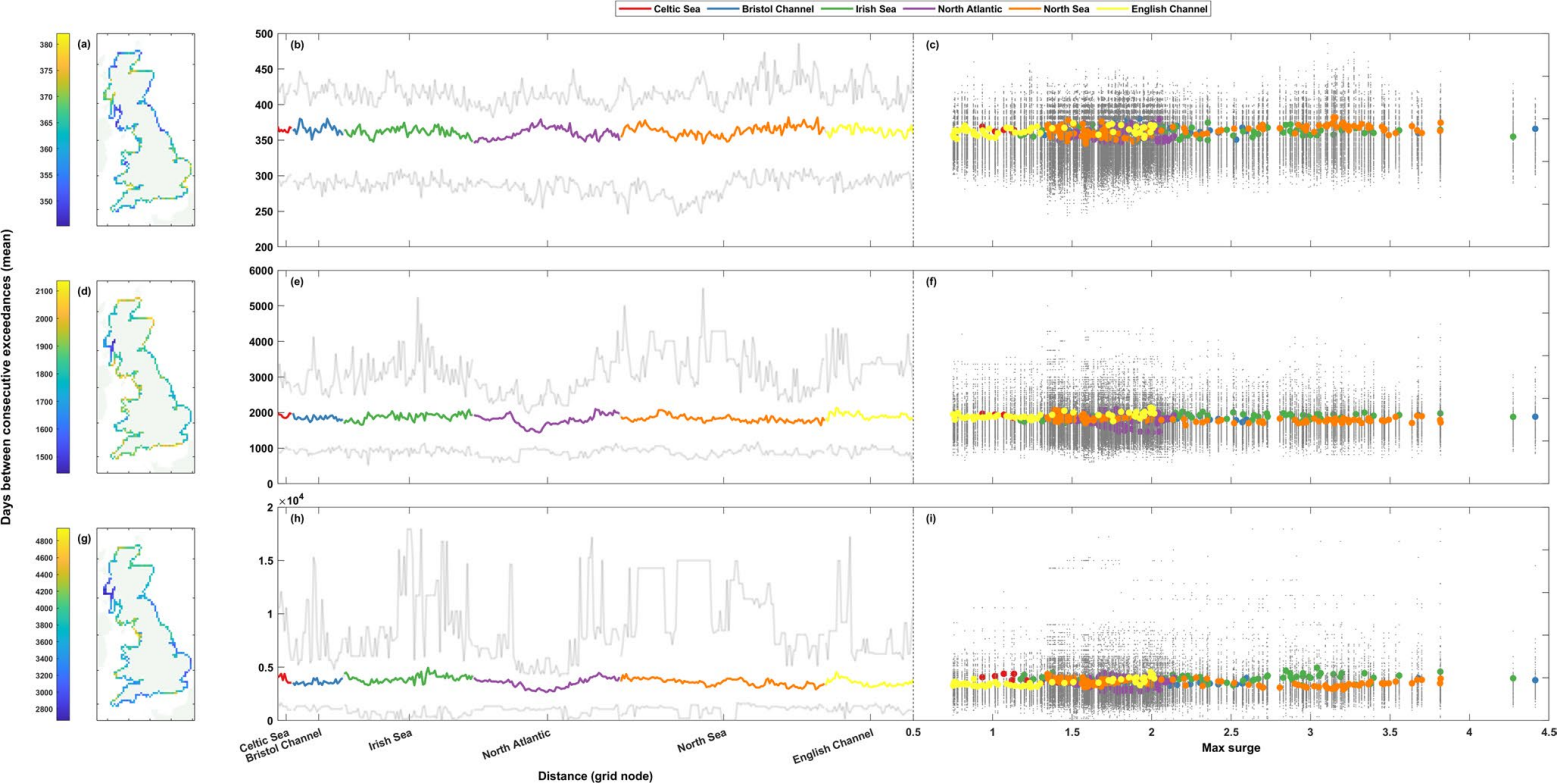
Mean number of days between consecutive non-tidal residual exceedances. The top row (**a**-**c**) shows results at the 1 in 1-year return level, the middle row (**d**-**f**) the 1 in 5-year return level, and the bottom row (**g**-**i**) the 1 in 10-year return level. Percentages are shown spatially in panels **a**, **d**, and **g** (left column), clockwise around the coast from the Southwest tip of England ending back at Newlyn in panels **b**, **e**, and **h** (middle column), and finally shown as a scatter plot against maximum non-tidal residual at that grid node in panels **c**, **f**, and **i** (right column). The grey lines (dots) in panels **b**, **e**, and **h** (**c**, **f**, and **i**) show the range of results from the 50-year rolling window analysis

**Fig. 6:**
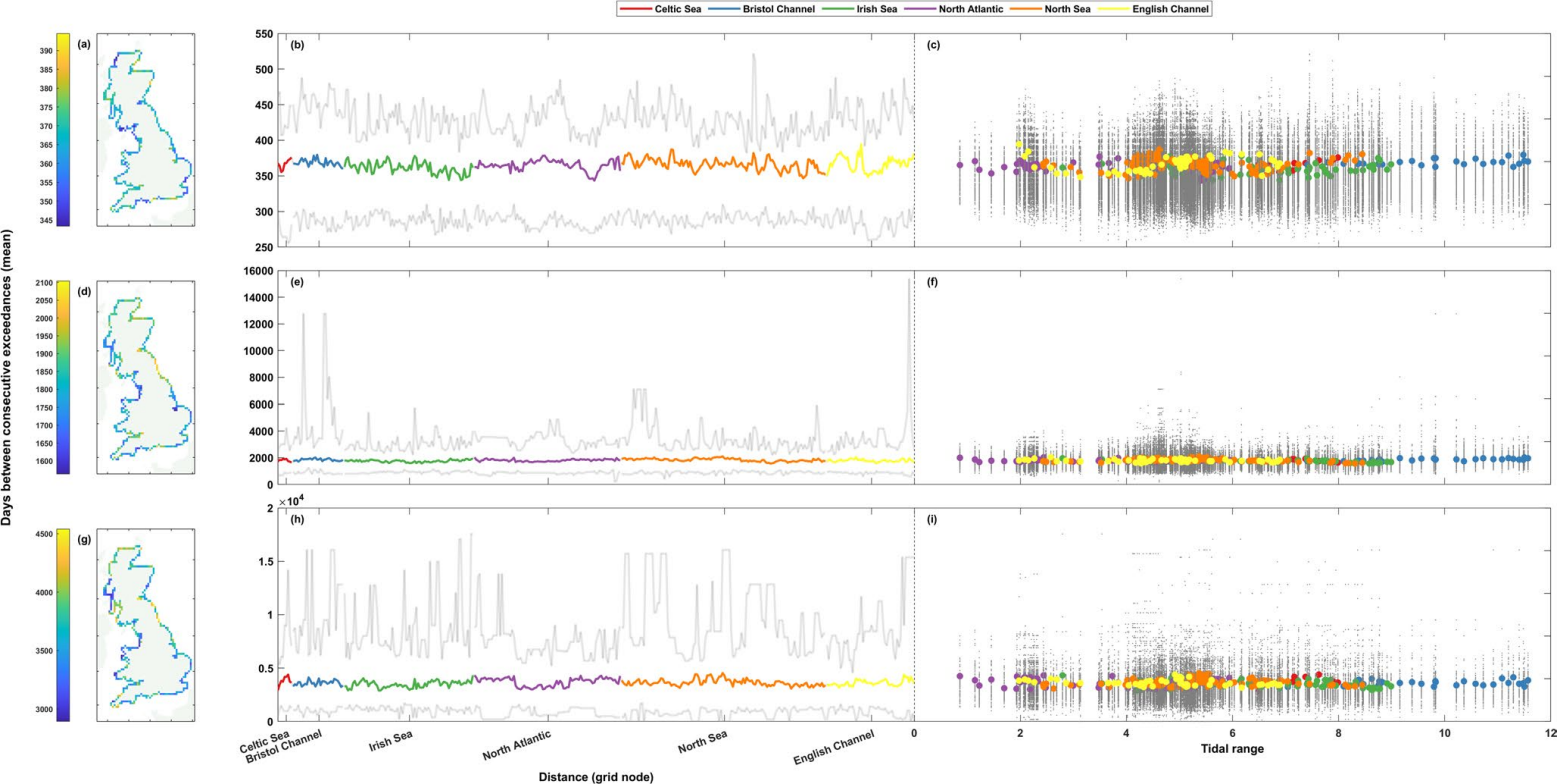
Mean number of days between consecutive sea level exceedances. The top row (**a**-**c**) shows results at the 1 in 1-year return level, the middle row (**d**-**f**) the 1 in 5-year return level, and the bottom row (**g**-**i**) the 1 in 10-year return level. Percentages are shown spatially in panels **a**, **d**, and **g** (left column), clockwise around the coast from the Southwest tip of England ending back at Newlyn in panels **b**, **e**, and **h** (middle column), and finally shown as a scatter plot against tidal range at that grid node in panels **c**, **f**, and **i** (right column). The grey lines (dots) in panels **b**, **e**, and **h** (**c**, **f**, and **i**) show the range of (all) results from the 50-year rolling window analysis

**Fig. 7:**
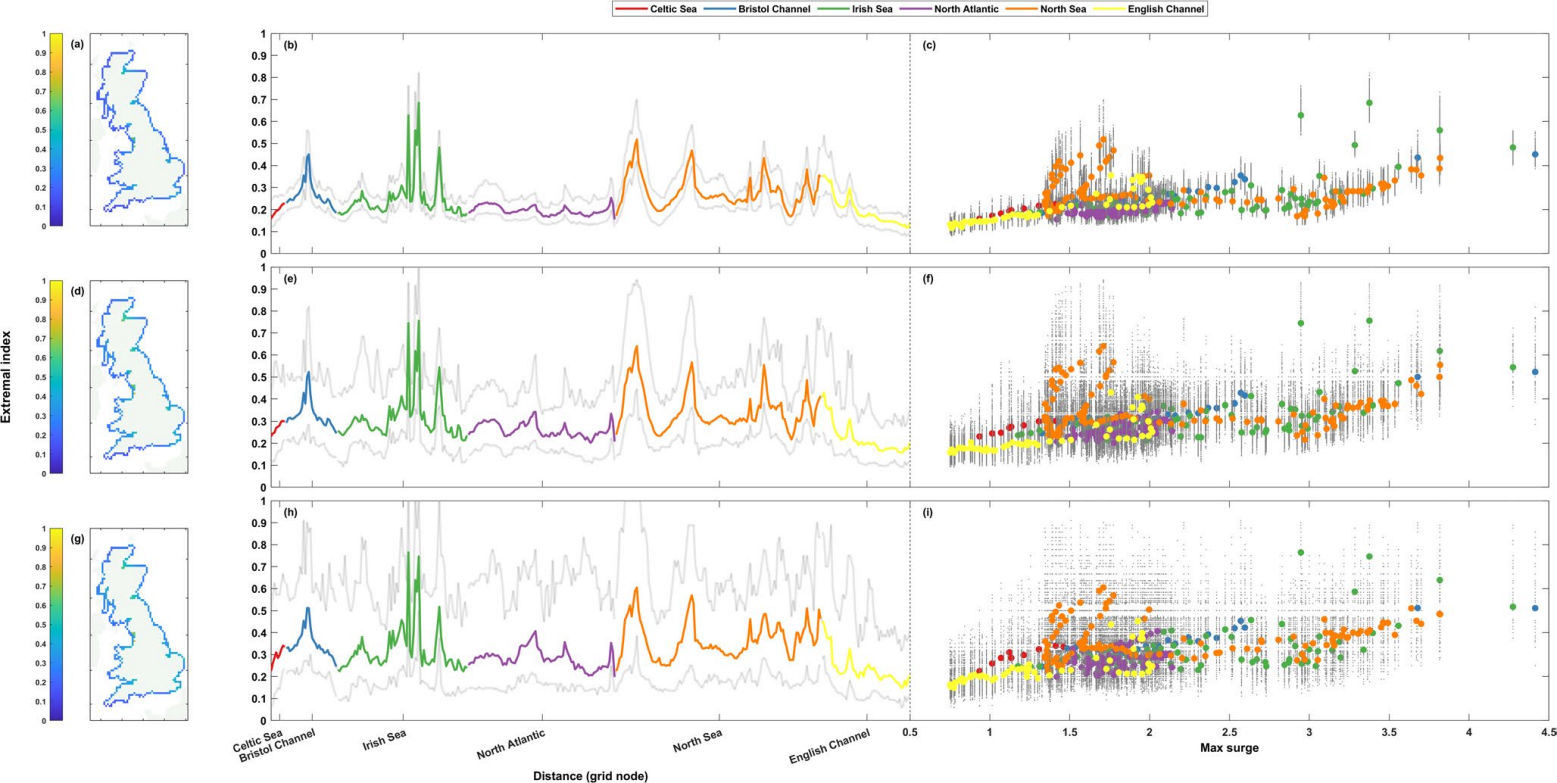
Extremal index (D-gaps) for non-tidal residual timeseries. The top row (**a**-**c**) shows results at the 1 in 1-year return level, the middle row (**d**-**f**) the 1 in 5-year return level, and the bottom row (**g**-**i**) the 1 in 10-year return level. Percentages are shown spatially in panels **a**, **d**, and **g** (left column), clockwise around the coast from the Southwest tip of England ending back at Newlyn in panels **b**, **e**, and **h** (middle column), and finally shown as a scatter plot against maximum non-tidal residual at that grid node in panels **c**, **f**, and **i** (right column). The grey lines (dots) in panels **b**, **e**, and **h** (**c**, **f**, and **i**) show the range of (all) results from the 50-year rolling window analysis

**Fig. 8:**
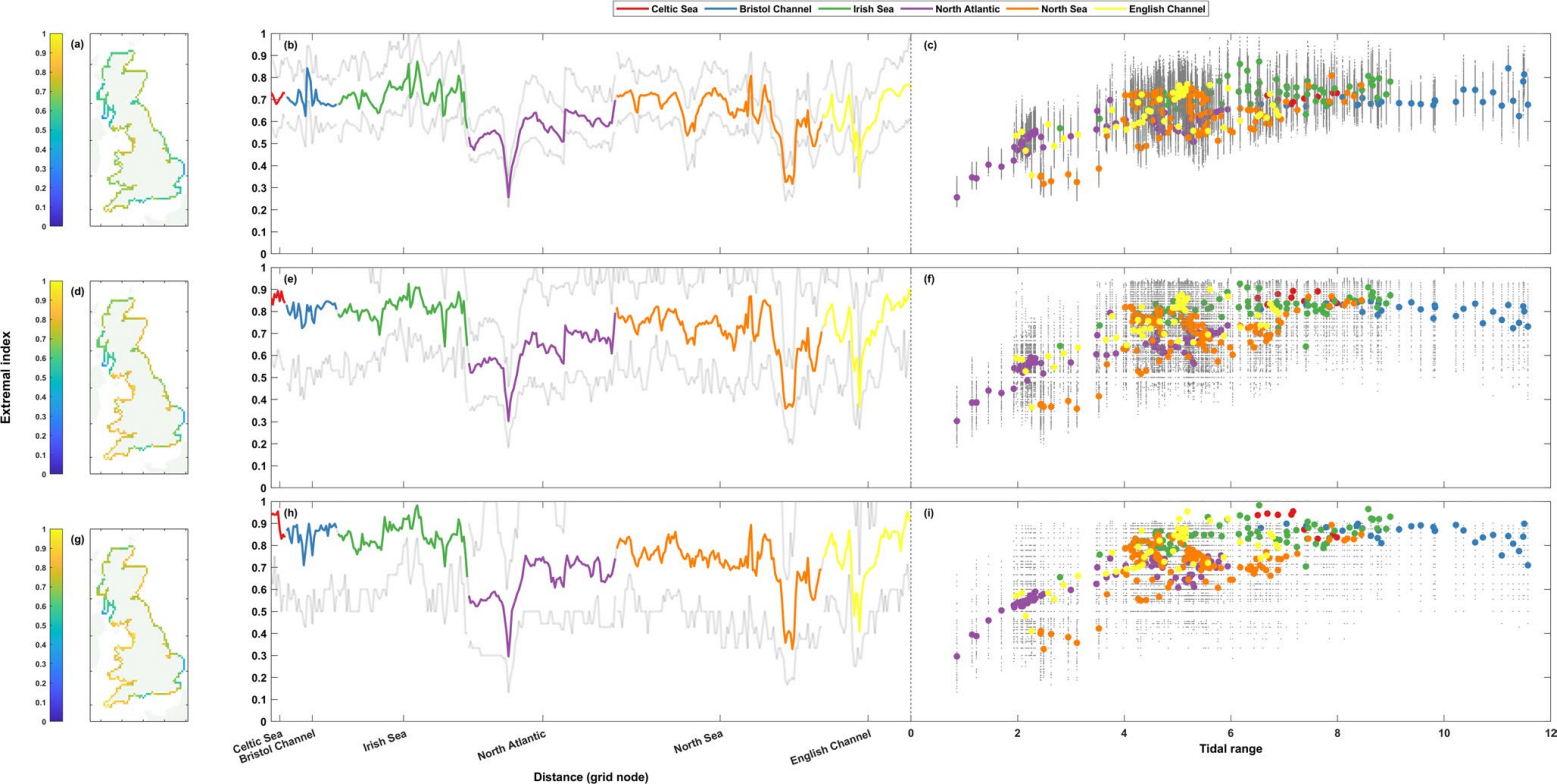
Extremal index (D-gaps) for sea level timeseries. The top row (**a**-**c**) shows results at the 1 in 1-year return level, the middle row (**d**-**f**) the 1 in 5-year return level, and the bottom row (**g**-**i**) the 1 in 10-year return level. Percentages are shown spatially in panels **a**, **d**, and **g** (left column), clockwise around the coast from the Southwest tip of England ending back at Newlyn in panels **b**, **e**, and **h** (middle column), and finally shown as a scatter plot against tidal range at that grid node in panels **c**, **f**, and **i** (right column). The grey lines (dots) in panels **b**, **e**, and **h** (**c**, **f**, and **i**) show the range of (all) results from the 50-year rolling window analysis

Figures 3, 4, 5, 6, 7 and 8 show the results for the clustering statistics at each return level, for sea levels and non-tidal residuals. In each figure, panels a-c (top row) show the 1 in 1-year return level results, panels d-f (middle row) the 1 in 5-year, and panels g-i (bottom row) the 1 in 10-year. Panels a, d, and g (left column) show the results spatially on a map of Great Britain, panels b, e, and h (middle column) show the results as a line around the coast starting from the Southwest corner of England, going clockwise around Great Britain finishing back at Newlyn. Panels c, f, and i (right column) show each grid nodes clustering statistic against tidal range for sea level, and against the maximum surge level for non-tidal residual. The line and scatter panels (b-c, e-f, h-i, middle column and right column) show the range of values, and all the values, respectively, from the rolling window analysis in grey. Results are shown against the tidal range or maximum surge level as one may expect to see a correlation between the clustering statistics and these values. Tides determine the majority of sea level exceedances in the UK, and therefore the clustering of these exceedances (Haigh et al. 2016; Jenkins et al. 2023), so a correlation between the tidal range and the clustering of sea level exceedances is logical. In a similar vein, areas that see larger surge values can be inferred to have a higher level of storminess, and with the formation of large storms in the North Atlantic creating favourable conditions for the formation of further cyclones (Priestley et al. 2020; Dacre and Pinto 2020), one could expect large surge values to correlate with the clustering of non-tidal residual exceedances.

#### Exceedance counts

First, we consider the interannual clustering, by analysing the percentage of storm years with a sea level or non-tidal residual exceedance. The lower the percentage, the higher the level of clustering as the exceedances occur in a less uniform temporal frequency throughout the timeseries. One could expect a 1 in 1-, 1 in 5-, and 1 in 10-year return level exceedance to be seen in around ~ 63%, ~ 18%, ~ 9.5% of storm years. Figure 3 shows the percentage of years with an exceedance for the non-tidal residual. The results for the whole timeseries do not show a significant spread of values, with the range in percentage of storm years with an exceedance only being around ~ 5–8% for the whole of the UK coastline at the return level thresholds. The results for all return levels show slightly lower percentages than what could be statistically expected, with the largest differences being seen at the lowest return level threshold. No clear relationship is shown with maximum surge values at any return level (Fig. 3c). Spatially, all regions see variations in the levels of clustering at every return level, but clustering is most prevalent (smallest values of percentage of storm years with an exceedance) for the lowest return level (Fig. 3a) in the North of Great Britain, particularly from the Kintyre Peninsula to the Northern coast of the Scottish Highlands, and then from Lerwick down to Aberdeen (< 56%). Relatively higher percentages (less clustering) (> 58%) are seen for the rest of the East coast of Great Britain and for most of England and Wales, with exceptions for Cornwall, the North coast of Wales, and parts of Cumbria, which see a higher prevalence of clustering (< 56%). At the higher 1 in 5-year return level (Fig. 3d), the Irish Sea sees the highest levels of clustering (< 17%) and the region surrounding Loch Linnie sees lower levels of clustering (> 19%) than the rest of Great Britain. This pattern also holds at the 1 in 10-year return level (Fig. 3g). The Northeast coast of England sees relatively higher levels of clustering (< 17%) at the 1 in 5-year return level than at the 1 in 1-year return level, where it saw relatively lower levels in relation to the rest of the coastline. However, as mentioned before, the variations in the levels of clustering calculated for the whole timeseries are relatively minor and the spatial differences noted above are in line with this. At the highest 1 in 10-year return level, the greatest degree of clustering (< 8.5%) is seen in Northern Scotland, and from the most Southwestern point of the Welsh coast stretching north through the Irish Sea to the Kintyre Peninsula. The lowest levels of clustering (> 10.5%) are seen, as mentioned, around Loch Linnie, but lower values are also seen from Looe to the Isle of Wight and at the Essex and Suffolk coasts.

Despite these minor variations in results for the entire timeseries, the rolling window calculations (Fig. 3b-c, e-f, and h-i) show that the percentage of storm years with an exceedance for any given 50-year period can vary between ~ 14–33%, ~ 10–24%, and ~ 8–18% at the 1 in 1-, 1 in 5-, and 1 in 10-year return level, respectively.

Figure 4 shows the repeat analysis for still sea level. The range in percentage of storm years with an exceedance is larger in spread than the non-tidal residual data, being around ~ 6–14% for the whole coastline across all return levels. The percentages around the coastline at the higher return levels (Fig. 4d-i) are similar in magnitude to the results from the non-tidal residual data (Fig. 3d-i) (results at the 1 in 5- and 1 in 10-year return levels being between ~ 14–20% and ~ 6–12%, respectively), but at the lowest 1 in 1-year return level the percentages of storm years with a sea level exceedance (Fig. 4a-c) see slightly more variation in magnitude (results between ~ 49–63%, compared to ~ 53–60% for non-tidal residual) and also have a higher level of spatial coherence around the coast with clearer regional signals. None of the return levels show any clear relationship between tidal range and percentage of storm years with a sea level exceedance (Fig. 4c, f, and h). For example, although the large tidal ranges of the Bristol Channel all see percentages less than 55% at the 1 in 1-year return level, percentages of equally low magnitude are also seen at Celtic Sea, English Channel and North Sea sites with far smaller tidal ranges. At the 1 in 1-year return level, the Southwest of England and the East coast of Scotland see the highest levels of clustering (Fig. 4a), whilst the lowest levels are seen on the West coast of Scotland and Cumbria, and the East coast of England. This pattern is not repeated at the higher 1 in 5-year return level (Fig. 4d), with the highest levels of clustering in the Southwest of England restricted to Cornwall, and the higher levels seen on the East coast of Scotland now extending down to the North York Moors. The lowest levels of clustering are seen in the Irish Sea, from Snowdonia up to Dumfries and Galloway, as well as in Lincolnshire and some adjoining parts of Norfolk. At the highest 1 in 10-year return level (Fig. 4g), the spatial pattern remains similar to that of the 1 in 5-year return level. However, the lowest levels of clustering seen in the Irish Sea from Snowdonia northwards stop around Lancaster, and the similarly low levels that were seen in Lincolnshire and adjoining parts of Norfolk are more akin to the nearby coastline. The areas that saw the highest levels of clustering remain largely the same.

Unlike the whole timeseries analysis, which saw slightly more variation in the magnitude of results for sea levels than for non-tidal residuals, the rolling window calculations see a remarkably similar level of variation in magnitude (Fig. 4b-c, e-f, and h-i). The percentage of storm years with a sea level exceedance for any given 50-year period varies by ~ 14–33%, ~ 10–24%, and ~ 6–16% at the 1 in 1-, 1 in 5-, and 1 in 10-year return level, respectively.

#### Days between events

Next, we consider the levels of intra-annual clustering by examining the mean number of days between consecutive exceedances at a given location. We first consider the results for non-tidal residual timeseries, shown in Fig. 5. The range in the mean number of days between consecutive exceedances around the coastline for the whole timeseries analysis is ~ 36, ~696, and ~ 2,288 days at the 1 in 1-, 1 in 5-, and 1 in 10-year return levels, respectively. Like with the percentage of storm years with an exceedance analysis, there is no apparent relationship between the clustering statistic and the maximum surge level recorded (Fig. 5c, f, and i). At the 1 in 1-year return level (Fig. 5a), the coastline has a lower level of spatial coherence than for the percentage of storm years with an exceedance analysis, with more variation between neighbouring grid nodes. The lowest values (< 350) of mean number of days between exceedances (higher level of clustering) are found from Dumfries and Galloway to the Kintyre Peninsula, from the Inner Sound to Wick, and from Aberdeen to the North York Moors. Some low values are also seen on the Welsh coastline around Snowdonia and Llŷn. Higher values (> 370) of mean number of days between exceedances (lower levels of clustering) are found on the English East and South coasts, from Hull to Bournemouth – but the relatively higher results seen along this stretch of coastline are variable and interspersed with some lower values that represent higher levels of clustering. Low values (< 350) are found across a couple of grid nodes on opposing sides of the Bristol Channel at Swansea/Gower and Exmoor. In Scotland, higher values are recorded on the West coast from Scarba to Loch Alsh, and on the North Sea from Helmsdale to Peterhead. The 1 in 5-year return level results (Fig. 5d) show some opposing patterns compared to the 1 in 1-year. For example at the 1 in 5-year return level, higher values (> 2000) are seen on the Northern tip of Scotland, Aberdeenshire, and in the Irish Sea from Barmouth to the Kintyre Peninsula, whilst lower values (< 1800) are seen from the North side of the Kintyre Peninsula up to Loch Alsh, all in direct contrast to the 1 in 1-year return level results. Some of the highest values at the 1 in 5-year return level are found in the English Channel, from Dungeness to Hengistbury Head. At the highest 1 in 10-year return level (Fig. 5g), the lowest values (< 3000) are again found around the Slate Islands to Loch Hourn. Lower values are also seen at large parts of the English North Sea coast, as well as on the English South coast from Bournemouth to Land’s End. Higher values are found on the Northern tip of Scotland, the North coast of Cornwall, the Pembrokeshire Coast, on the South coast by Eastbourne, and finally the highest values (> 4400) are seen at the Irish Sea coast from Blackpool to the Lake District.

The rolling window analysis shows that in any given 50-year period, the mean number of days between consecutive non-tidal residual exceedances at a grid node can vary by ~ 88–191, ~ 986–4757, and ~ 2871–17,793 days at each of the 1 in 1-, 5-, and 10-year return levels, respectively.

The mean number of days between consecutive extreme sea level exceedances is shown in Fig. 6. Around the coast, the mean number of days between consecutive sea level exceedances ranges by up to ~ 51, ~541, and ~ 1648 days at the three return level thresholds, with the average number of days being 365, 1809, and 3619. No clear relationship is seen at any return level threshold between the mean number of days between consecutive sea level exceedances and tidal range (Fig. 6c, f, and i). Spatially, there are lower values (< 355) of mean days between consecutive 1 in 1-year sea level exceedances (Fig. 6a) in the Irish Sea, the Southeast coast of England (Lincolnshire Wolds to Rye Harbour), the South coast of England from Worth Matravers to Dartmouth, and then on the West coast of Scotland from Loch Ewe to Bettyhill. Higher values (> 380) are seen in Scotland from Strathy to Stonehaven, and then on the South coast of England from Eastbourne to Littlehampton and Milford on Sea to Swanage. The higher 1 in 5-year return level (Fig. 6d) sees higher values (> 1950) in the Bristol Channel, the North of Scotland, and particularly on the North Sea coast from Edinburgh to Flamborough. Lower values (< 1650) are seen again in the Irish Sea (Solway Coast to the Llŷn Peninsula), as well as the in the Southeast coast of England from Fraisthorpe to Worthing, and on the South coast from Kimmeridge to Mevagissy. At the highest 1 in 10-year return level (Fig. 6g), the spatial pattern is broadly similar to the 1 in 5-year return level. There are lower values (< 3250) again in the Southeast coast of England from Skegness to Portsmouth, and in the Irish Sea but with the lowest values starting slightly further south than at the 1 in 5-year return level – from Lancaster to the Llŷn Peninsula. Relatively lower values are also seen at the Pembrokeshire Coast. Opposing signals are seen on consecutive parts of the West coast of Scotland, with high values (> 4000) in the Firth of Clyde and on the South and East coasts of the Kintyre Peninsula, but low values (< 3250) are seen on the West coast of the Kintyre Peninsula, then continuing up to the Small Isles.

The mean number of days between consecutive sea level exceedances at a grid node varies by ~ 84–231, ~ 1290–14,780, and ~ 3140–16,809 days in the rolling 50-year window analysis, at the 1 in 1-, 5-, and 10-year return levels, respectively.

#### Extremal index

Finally, we evaluate clustering throughout the entire record by calculating the extremal index, $\:\theta\:$. Figure 7 shows the results of the extremal index calculations for the non-tidal residual timeseries. The $\:\theta\:$ results for all three return levels for the entire timeseries are almost identical. Most values do not deviate far from the average of all the grid nodes, but a few grid nodes have far larger results than the rest. The spatial plots (Fig. 7a, d and g) show that these grid node locations are typically located at shallow water, estuarine locations, such as the Moray Firth, the Firth of Forth, the Solway Firth, Morecombe Bay, the Ribble and Alt Estuaries, the Seven Estuary, the Wash, and the Thames Estuary. The lowest $\:\theta\:$ values across the three return levels are all found on the South coast of England, from Worth Matravers to St. Ives. There is a slight positive association between maximum surge level and the extremal index (Fig. 7c, f, and i), with most of the values following this association. The deviations from this trend are again all results from the shallow water, estuarine locations highlighted prior that have far greater $\:\theta\:$ values than the other grid nodes.

The rolling window analysis shows that for any given 50-year period at a coastal grid node, the range of non-tidal residual extremal index values match the pattern of the results for the whole timeseries. However, at every increase in return level, the range in $\:\theta\:$ increases, from ~ 0.06–0.31, ~ 0.16–0.6, and ~ 0.24–0.79 although the pattern remains largely the same.

The extremal index calculations for the sea level timeseries are shown in Fig. 8. Like for the non-tidal residual timeseries, the $\:\theta\:$ results for sea level see similar patterns at all three return level thresholds, although there is more distinction between the sea level results. The average $\:\theta\:$ value increases slightly with each return level increase (~ 0.65, ~ 0.72, and ~ 0.75, respectively), with most regions (Fig. 8a-b, d-e, and g-h) seeing the majority of their grid nodes having minor increases in the $\:\theta\:$ values that mirrors this increase in the average. However, interestingly, the grid nodes that have the lowest values at the 1 in 1-year return level (Fig. 8a-c) maintain these low values at each return level, seeing virtually no change in magnitude despite the slight increases elsewhere. These regions are the Kintyre Peninsula, the Suffolk coastline, and the Isle of Purbeck. The highest $\:\theta\:$ values are seen on the Southwestern and Western coast of England (Fig. 8a, d, and g), from Dartmouth stretching up to Scotland at Dumfries and Galloway. There is a positive association with $\:\theta\:$ and tidal range (Fig. 8c, f, and i), with the only significant deviations from the general trend being some of the grid nodes at the Suffolk coast and the Isle of Purbeck that see lower $\:\theta\:$ values in relation to their tidal range when compared to the rest of the coastline.

Similar to the non-tidal residual extremal index results, the rolling window calculations of the extremal index for sea levels show a comparable pattern to that of the overall timeseries, again just with a larger range at each increase in return level threshold.

### Measured and modelled data comparison

**Fig. 9:**
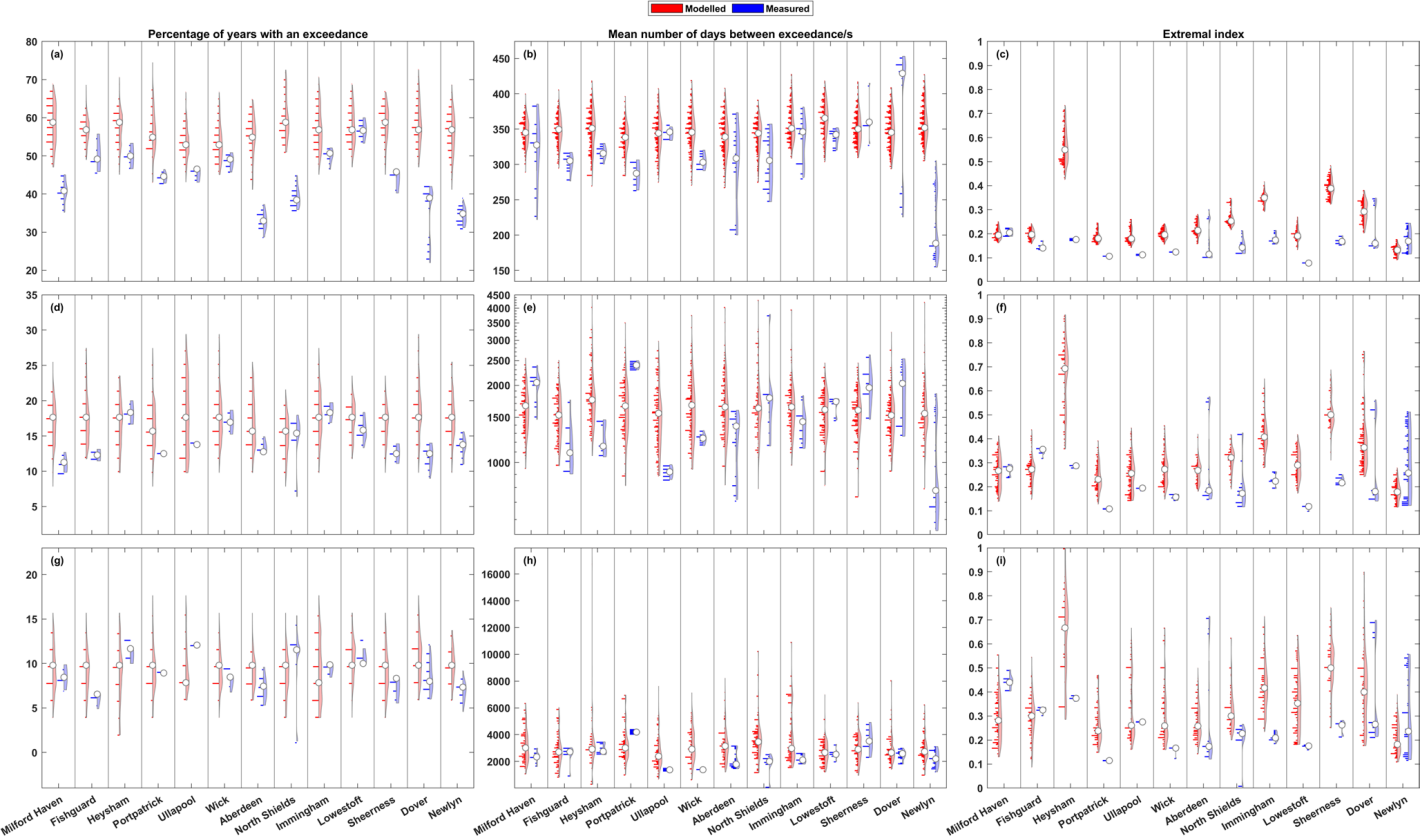
Comparison of measured and modelled non-tidal residual results from the 50-year rolling analysis. Each pair of violins represent the UKNTGN tide gauge record (right violin, blue) and the nearest model grid node timeseries (left violin, red). The top row (**a**-**c**) shows results at the 1 in 1-year return level, the middle row (**d**-**f**) the 1 in 5-year return level, and the bottom row (**g**-**i**) the 1 in 10-year return level. The left column (**a**, **d**, and **g**) shows the percentage of storm years with an exceedance, the middle column (**b**, **e**, and **h**) shows the mean number of days between consecutive non-tidal residual exceedances, and the right column (**c**, **f**, and **i**) shows the extremal index (D-gaps)

**Fig. 10:**
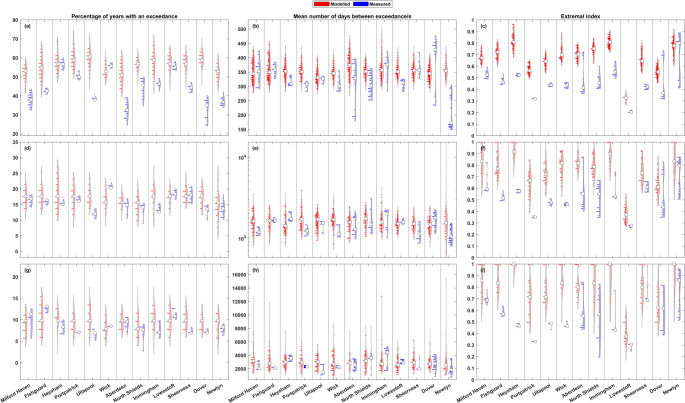
Comparison of measured and modelled sea level results from the 50-year rolling analysis. Each pair of violins represent the UKNTGN tide gauge record (right violin, blue) and the nearest model grid node timeseries (left violin, red). The top row (**a**-**c**) shows results at the 1 in 1-year return level, the middle row (**d**-**f**) the 1 in 5-year return level, and the bottom row (**g**-**i**) the 1 in 10-year return level. The left column (**a**, **d**, and **g**) shows the percentage of storm years with an exceedance, the middle column (**b**, **e**, and **h**) shows the mean number of days between consecutive sea level exceedances, and the right column (**c**, **f**, and **i**) shows the extremal index (D-gaps)

Next, we consider the rolling window results against that of their observed data counterparts. By comparing results from 50-year periods in the modelled and observed data, we can assess how well the model is capturing the levels of clustering seen at the Great British coast. The comparison in rolling window clustering statistics between the 13 longest UKNTGN tide gauge records and the closest grid nodes in the model is shown in Fig. 9 for non-tidal residual and Fig. 10 for sea level. In both figures, panels a-c (top row) show results at the 1 in 1-year return level, panels d-f (middle row) the 1 in 5-year, and panels g-i (bottom row) the 1 in 10-year. The left column (panels a, d, and g) shows the percentage of years with an exceedance, the middle column (panels b, e, and h) shows the mean number of days between consecutive exceedances, and the right column (panels c, f, and i) shows the extremal index. Each individual panel has the results for the longest UKNTGN tide gauge records and their nearest modelled grid node timeseries in turn. The red violin shows the modelled data and the blue violin shows the observed/measured data. The right side of the violins show a kernel density estimate for the rolling window results, and the left side of the violins show a histogram of the rolling window results that represents the relative amount of data points at that magnitude. The central circular marker shows the median of the results.

#### Exceedance counts

The comparison of the rolling window percentage of storm years with an exceedance for measured and modelled data is shown in Fig. 9a, d, and g for non-tidal residual and Fig. 10a, d, and g for sea level. For the percentage of storm years with a non-tidal residual exceedance, the largest differences are seen at the 1 in 1-year return level (Fig. 9a). Of the 13 sites with the longest records, only three sites (Heysham, Immingham and Lowestoft) have their range of percentage of storm years with a non-tidal residual exceedance calculated from the rolling window analysis completely within the range of their modelled counterparts. All other sites see their range of values extend below, or be completely below, (lower percentage value) the range of the results from the nearest modelled grid node. Milford Haven, Aberdeen, North Shields, Dover and Newlyn all have their range of percentages of storm years with a non-tidal residual exceedance sitting outside of the range of the results from the nearest modelled grid node by between ~ 2–10%. At the higher 1 in 5-year return level (Fig. 9d), none of the measured sites see their range of values sit wholly outside the modelled grid nodes, and just three sites (Fishguard, North Shields and Dover) see their lowest values extend further than their modelled grid node equivalents. At the highest 1 in 10-year return level (Fig. 9g), there are fewer measured results to work with, and thus there is a lower spread of values in the measured results. Again, all but three sites see their measured results extend past the modelled range of results, those being Aberdeen, North Shields, and Newlyn, whose lowest values extend lower than the range of the counterpart modelled grid nodes. The lower percentage values seen in some of the measured results imply higher levels of clustering.

Figure 10a, d, and g shows the percentage of storm years with a sea level exceedance. At the 1 in 1-year exceedance level (Fig. 10a), the modelled data tends to see a slightly greater spread of values than for the modelled non-tidal residual. All the measured tide gauge sites bar Wick have median values that are below their modelled grid node equivalents. Of these, Ullapool and Dover have all their values be less than the minimum percentage from their modelled grid node counterparts, with Aberdeen almost the same, only overlapping by ~ 0.15%. Dover sees the largest difference, with a ~ 5% gap between the maximum measured value and the minimum modelled value. The 1 in 5-year return level (Fig. 10d) sees more uniformity across the results from the different sites. Although only three measured median values are higher than their modelled grid node equivalents, most medians are within ~ 2% of their equivalent and no measured site has its entire range of values sit outside the modelled range of values. Only two sites (Ullapool and Dover) have any measured percentage values that are lower than the minimum modelled value for their corresponding nearest grid node. The highest 1 in 10-year return level (Fig. 10g) sees even more uniformity across the modelled data, with all modelled median values bar Wick and North Shields being near identical. Like the 1 in 5-year return level, there are no large differences between the measured and modelled median percentages, and only North Shields has any measured values that are outside (in this case, below) the range of the matching grid node percentage values.

#### Days between events

The comparison of days between consecutive exceedances between the rolling windows for the measured gauge records and the modelled grid nodes is shown in Fig. 9b, e, and h for non-tidal residual and Fig. 10b, e, and h for sea level. For the mean number of days between consecutive non-tidal residual exceedances, as expected, there is a higher spread of values for both the measured and modelled data. Across the return levels, only Newlyn at the 1 in 1-year threshold (Fig. 9b) has almost all the measured range of values sitting outside the modelled grid node range of values. At their closest, there is less than a day between the values, but at their furthest there is ~ 273 days, with the median for measured and modelled data at Newlyn being 188 and 352 days, respectively – constituting a significantly higher level of clustering in the measured data. Seven other sites see their lower values having a smaller magnitude than the lowest values of their modelled counterparts: Milford Haven, Fishguard, Portpatrick, Aberdeen, North Shields, Immingham and Dover. Conversely, Dover is the only site that has values that are of a greater magnitude than the range of its modelled grid node. The spread of values at Dover are divergent, with a proportion being greater than almost all other results across all sites at the 1 in 1-year return level, and the other proportion from Dover being some of the lowest values seen at any tide gauge. The 1 in 5-year return level (Fig. 9e) sees Sheerness having the only measured values at a tide gauge being of a larger magnitude than the modelled equivalents, but 5 sites have results that are lower than that of the respective modelled data –of which, only the differences at Newlyn and Aberdeen are of significance (~ 253 and ~ 100 days between the measured and modelled minimum results, respectively). At the highest 1 in 10-year return level (Fig. 9h), only North Shields has measured days between consecutive exceedances results lower than its modelled counterpart’s lowest value of (~ 1% and ~ 4%, respectively). North Shields lowest value is ~ 32 days, with the modelled grid node nearest seeing a minimum of ~ 1162 days. All other sites’ values are within the ranges of their modelled grid node equivalents.

The mean number of days between consecutive 1 in 1-year sea level exceedances (Fig. 10b) sees a greater spread of modelled results than that of non-tidal residual exceedances, but with a similar overall pattern. Again, Newlyn is the only site that has nearly all of its measured values sit lower than the minimum modelled value for its nearest grid node. The results for Dover are once again divergent, with some of the highest values seen across all measured and modelled data, and values that are some of the lowest seen, being up to ~ 50 days lower than the lowest modelled value at the nearest grid node and ~ 247 days lower than the highest results for measured data at Dover. Bar Newlyn and Dover, all measured median number of days between consecutive sea level exceedances are within the range of modelled values. The other sites whose spread of measured values extend lower than their modelled counterparts are Wick, Aberdeen, North Shields, Immingham and Lowestoft. At the 1 in 5-year return level (Fig. 10e), only Sheerness has any measured values that are lower, and North Shields is the only site that has measured values that are higher, than their modelled grid node equivalent values. The highest 1 in 10-year return level (Fig. 10h) sees the modelled grid node results have far larger spreads/ranges than the measured tide gauge results. However, Ullapool, North Shields, Dover, and Newlyn still have measured values that are lower than any modelled value at their respective grid nodes. Eight sites have measured medians that are smaller than their modelled medians.

#### Extremal index

The comparison of extremal index values from the rolling window analysis is shown in Fig. 9c, f, and i for non-tidal residual and Fig. 10c, f, and i for sea level. The extremal index results for non-tidal residual show significant differences between the measured and modelled data at the 1 in 1-year return level (Fig. 9c). Eleven of the thirteen sites have the measured median value sitting lower than the range of modelled values, with seven of the sites having all measured values sitting lower. Milford Haven and Newlyn are the exceptions, Newlyn being the only site where both the measured median (~ 0.17) is higher than the modelled median counterpart (~ 0.13) and the measured values extend above the range of modelled values (~ 0.24 and ~ 0.18, respectively). The largest difference is seen at Heysham, where there is a difference of ~ 0.25 between the measured and modelled values, with the modelled values being the largest in magnitude and in range of all the sites. Aberdeen and Dover have a level of divergence, with large gaps between their minimum and maximum measured values, with few data points in the middle of the distribution. The 1 in 5-year return level (Fig. 9f) has a larger spread of extremal index results, particularly in the modelled data. Despite this, seven sites still have the median from the measured data sit below the range of modelled extremal index values. Five sites (Heysham, Portpatrick, Immingham, Lowestoft and Sheerness) have all measured values sit below the range of modelled values. Milford Haven, Fishguard and Newlyn all have a measured median that is higher than their modelled equivalent. Newlyn has measured values that are greater than any of the modelled results, as do Aberdeen and North Shields which show divergence in their results. Heysham also has the largest modelled values at this return level. At the highest 1 in 10-year return level (Fig. 9i), there is an increase in the spread of modelled values. Nine of the sites have the measured median extremal index below the modelled grid node counterpart, Milford Haven, Fishguard, Ullapool and Newlyn being the exceptions. Portpatrick and Lowestoft have all their measured values be of a lower magnitude than any of the modelled extremal index values at their nearest grid node.

Across all sites and return level thresholds, the measured and modelled extremal index medians for sea level (Fig. 10c, f, and i) are greater than their non-tidal residual counterparts, with there also being a tendency for a greater difference between the measured and modelled sea level extremal index results than there were for the non-tidal residual. At the 1 in 1-year return level (Fig. 10c), only one site (Newlyn) has a measured median that is greater than its modelled equivalent, with all other measured medians being outside the range of modelled values at their site. Nine of the thirteen sites have all their measured values be smaller than any of the modelled values at the nearest grid node. The 1 in 5-year return (Fig. 10f) level has a general trend of larger extremal index modelled values and increased spreads of modelled values. This is not apparent in the measured data, with Fishguard, Heysham, Portpatrick, Ullapool, Wick and Lowestoft seeing virtually no difference in the magnitude or spread of values, and only Milford Haven and Aberdeen see a significant difference in spread. All modelled medians are greater than their measured counterparts, with seven measured medians being smaller than all the modelled values at their given sites. At the 1 in 10-year return level (Fig. 10i), both the measured and modelled results are very similar to the lower 1 in 5-year return level, the only differences being some sites seeing an increase in spread and a couple of sites having a greater median value.

## Discussion

Previous assessments of clustering of extreme sea levels and non-tidal residuals (e.g., surge) have typically focused on measured datasets. However, measured datasets are limited by their variation in length with few longer records, data gaps, and lack of spatial coverage, all of which makes it hard to carry out a detailed assessment of clustering and difficult to examine spatial patterns around coastlines, compare between sites and come to robust conclusions. On top of this, the influence of climate change on recent decades makes it hard to decipher if patterns in clustering seen are effects of this climatic change, or to be expected with natural variability. Here, we have used a near 500-year modelled dataset of sea level and non-tidal residual under pre-industrial conditions and have quantified the level of temporal clustering seen around Great Britain under natural variability. Without clustering, one could expect a 1 in 1-year exceedance event to occur in ~ 63% of storm years, a 1 in 5-year exceedance event to occur in ~ 18% of storm years, and a 1 in 10-year exceedance event to occur in ~ 9.5% of storm years. One could particularly expect to see results that would exactly mirror their statistical expectancy when dealing with large amounts of data, like here, where there are near-500 years of hourly data. We show that sea level and non-tidal residual extremes occur in a slightly smaller percentage of years than what could be expected statistically, and that this difference is greatest at the smaller magnitude return levels. The average non-tidal residual and sea level percentage for all the Great British coastal grid nodes are around ~ 7% lower at the 1 in 1-year return level, ~ 1% lower at the 1 in 5-year return level, and ~ 0.5% lower at the 1 in 10-year return level than could be statistically expected. This is consistent with Jenkins et al. (2023), where higher magnitude events, as expected, cluster less than lower magnitude events, but lower magnitude events also cluster far more than could be expected statistically in the UK observed record. The mean number of days between exceedances also average to relatively near their statistical expectancies for all parameters and return levels.

Despite clustering parameters often averaging near their statistical expectancy over the near 500-year timeseries, we show that there is significant multi-decadal variation in clustering across all return level thresholds. By employing a 50-year rolling window to repeat our analysis, we highlighted the difference in the level of clustering seen at a given location when selecting a timeframe of the average data length of a tide gauge in the UKNTGN. Clustering of both sea levels and non-tidal residuals is far more apparent, with some parts of the coastline recording a percentage of storm years with an exceedance value as low as ~ 35%, ~ 5%, and ~ 2%, and rising as high as ~ 76%, ~ 33%, and ~ 21% at each return level threshold, respectively – showing the large differences in clustering seen in different 50-year periods, with this mirrored in the mean days between consecutive exceedances results. For example, the 1 in 5-year return level for sea levels is a commonly used threshold in coastal research in the UK as it excludes high magnitude sea levels that are caused exclusively by high tides. We show that in the data used here, that is the best representation of natural variability, the mean number of days between 1 in 5-year sea level exceedances in a period that is the average length of a UKNTGN tide gauge, can vary by up to 14,780 days. Therefore, it is concluded that clustering levels that are calculated from the shorter UK measured records may be heavily biased by the large multi-decadal variability.

The results for the extremal index stand apart from the other clustering statistics and highlights the problem in using different descriptors of clustering, as although they all intend to synthesise clustering into single values, the methods of doing so can produce differing results. There is no statistical expectancy for the extremal index, and surprisingly, in direct contrast to the other parameters, little variation is seen in the extremal index results at the different return level thresholds, yet relatively large differences are seen spatially around the coastline. It is also the only parameter that shows any relationship between sea level clustering and tidal range, and non-tidal residual clustering and max surge. However, it is unclear why variations in the non-tidal residual results are only seen at estuarine or shallower water locations where large increases in $\:\theta\:$ are seen. It is possible that the model struggles to get the tide, surge, and interaction between the two correct in areas where the geometry of the coast is not suitable for the model resolution. With these results being so different to the other clustering parameters, and with them not being able to be statistically grounded with what one would expect, it is difficult to assess whether these results accurately portray clustering of sea level and non-tidal residual exceedances for the model timeseries alone. They are therefore not discussed in great detail here and further research into the utility of the extremal index in this context would be of interest.

Our rolling window comparison of the model against the longest UK records shows that the model tends to underestimate the levels of clustering seen in the observed record across all clustering parameters and return levels. There is significant multi-decadal variability seen in the measured results, as seen in the model and discussed above, but the values for measured records tend to be of a smaller magnitude, which indicates a greater level of clustering. In many cases, the measured results see their range of values sit completely, or nearly completely, outside of the lowest modelled value for a given clustering parameter. This suggests the model is not exhibiting the level of clustering seen in the real world. Further research into the modelling of mid-latitude storms and their clustering is required to more accurately model the footprints of those storms when they are realised at the coast as extreme sea levels and storm surges. The measured tide gauge records had MSL trends removed, but climate change was present, so future research into whether recent decades see higher levels of clustering would also be of interest, to determine either an underestimation in the model, or a climate change signal causing higher clustering. This is of particular interest in the context of a projected increase in the temporal clustering of storms over Europe by the end of the century (Chan et al. 2023).

Overall, and somewhat surprisingly, there was a lack of spatial coherence seen in the results (discarding the extremal index). In the observational record, Haigh et al. (2016) recorded spatial footprints for sea level extremes, and Jenkins et al. (2023) noted levels of spatial coherence in the observed record for the temporal clustering of sea level and surge extremes, but this spatial coherence is not seen to the same extent in this research, which uses modelled data. The spatial patterns seen here tend to be of no greater significance than the magnitude of difference between coastal grid nodes within a region, and any patterns that do appear often change at each return level analysed. It is uncertain why the spatial coherence does not mirror that of the observed record. As the modelled data record is so long, it could be expected that the relative magnitude of change around the coastline would be small, as the clustering statistics for the grid nodes may well average out to similar results over such a long record, even if the actual manner of clustering within the timeseries may differ, which is what we see in the rolling window analysis. However, it could still be anticipated that regional patterns would be more apparent even if the magnitude of change were small. It is unclear why the model does not see this level of spatial coherence. There are also some curious results from the model in coastal areas of complex topography, for example the Kintyre Peninsula often has notable characteristics throughout the analysis, and the extremal index results for non-tidal residuals seemed to be skewed by shallow water, estuarine locations. The extremal index results cannot be explained by the additional input of fluvial processes as they are not present in the model, instead they are likely a result of either the change of depth or coastal geometries not being properly resolved due to the model resolution.

In this research, we show that the clustering of extreme sea levels and non-tidal residuals vary significantly in different simulated 50-year periods, but we do not explore the drivers of such variation. Changes in large-scale ocean atmosphere patterns have been linked to increased/decreased storminess and cyclone clustering in the North Atlantic (Dacre and Pinto 2020), as well as sea level, storm surge and wave extremes in the United Kingdom (Woodworth et al. 2007; Malagon Santos et al. 2017). There has also been active research into the specific physical mechanisms that drive the clustering of extratropical storms (Priestley et al. 2020). It would be of great interest for future research to link the footprints of storms in the ocean (extreme sea levels, storm surges, and waves) to large-scale atmospheric patterns, but also to include the specific physical mechanisms of storm clustering in the atmosphere, such as Rossby wave breaking and secondary cyclogenesis. This could provide invaluable insight for natural hazard forecasting and preparedness action.

As Jenkins et al. (2023) noted, models often struggle to accurately represent sea level and surge extremes, but are the only option for gaining continuous, gap-free data of considerable time lengths around the coast. Our comparison of the longest UKNTGN gauges and their nearest model grid nodes presents significant differences in the results for the measured and modelled data, as mentioned above. This complicates the ability to generalise or project modelled results onto real world scenarios. However, the model used here is one of the best representations of our ability to model sea level and surge parameters currently available, being one of the well-validated operational storm surge forecasting models (Horsburgh et al. 2021), and forced with pre-industrial climatic conditions representing natural variability. As such, the results presented here are the current best estimate of what to expect, and as the modelled data tended to underestimate the levels of temporal clustering, these results can be considered as the minimum levels of clustering to be anticipated around the coastline of Great Britain.

## Conclusions

In this study we examined the levels of temporal clustering of extreme sea levels and storm surges seen under natural variability around Great Britain. To do this, we used a near 500-year model of sea levels and storm surges from the CS3 coastal shelf sea model forced with pre-industrial conditions from HadGEM3-GC3-MM. We used the clustering statistics of the percentage of storm years with an exceedance, the mean number of days between consecutive exceedances, and the extremal index to highlight clustering on interannual scales, intra-annual scales, and the timeseries as a whole. We then applied a 50-year rolling window and repeated the analysis to see the differences in the results obtained when focusing on a time period that is similar to the average length of a tide gauge in the UKNTGN.

We show that the first two clustering statistics, when applied to such long timeseries, can tend to produce values near their statistical expectancy. They also showed a low amount of variation around the coast. However, when a rolling window is applied, the results vary significantly with temporal clustering then becoming very apparent, with changes in the number of exceedance events and the amount of time between them for the entirety of the coastline. For example, at the three return level thresholds (1-, 5- and 10-year) respectively, the mean number of days between consecutive sea level or non-tidal residual exceedances at a locality can vary by ~ 231, ~14,780, and ~ 17,793 days depending on the 50-year time period analysed. No clear spatial pattern of clustering is seen, however, as regional signals of clustering change between the statistics, and often between changes in return level threshold. Importantly, the model does not appear to simulate the levels of clustering that are present in the measured record. The temporal clustering of sea levels and non-tidal residuals may be more frequent phenomena than current modelling best efforts suggest.

The temporal clustering of sea levels and storm surges present consecutive hazards that can amplify potential damages to both the natural and human elements of the coastal protection system. This research shows that in Great Britain, such clustering is prevalent around the coastline and seen over the entirety of long synthetic datasets. Under natural variability, significant variation in the levels of clustering is seen over interannual and intra-annual timescales – the results highlight the difficulty in characterising the level of temporal clustering in a given location when focusing on relatively short timeframes such as the average record length of a tide gauge in the UKNTGN. As mean sea levels rise, the number of extreme sea levels and storm surges at any magnitude will increase due to this increase in base level. When combined with the high levels of temporal clustering that can occur under natural variability shown here, significant numbers of storms could affect the coastline of Great Britain in quick succession.

## Supplementary Information

Below is the link to the electronic supplementary material.

Supplementary Material 1

## Data Availability

No datasets were generated or analysed during the current study.
