# Supplementary material for: Assessing the temporal clustering of coastal storm tide hazards under natural variability in a near 500-year model run
Source: Ocean Dyn. 2026 Feb 9;76(2):17. doi: 10.1007/s10236-025-01766-4 (PMC12883521; doi:10.1007/s10236-025-01766-4)
Supplement: Supplementary file 1 — Supplementary Material 1 [file 10236_2025_1766_MOESM1_ESM.docx]

Supplementary material

**Assessing the temporal clustering of coastal storm tide hazards under natural variability in a near 500-year model run**


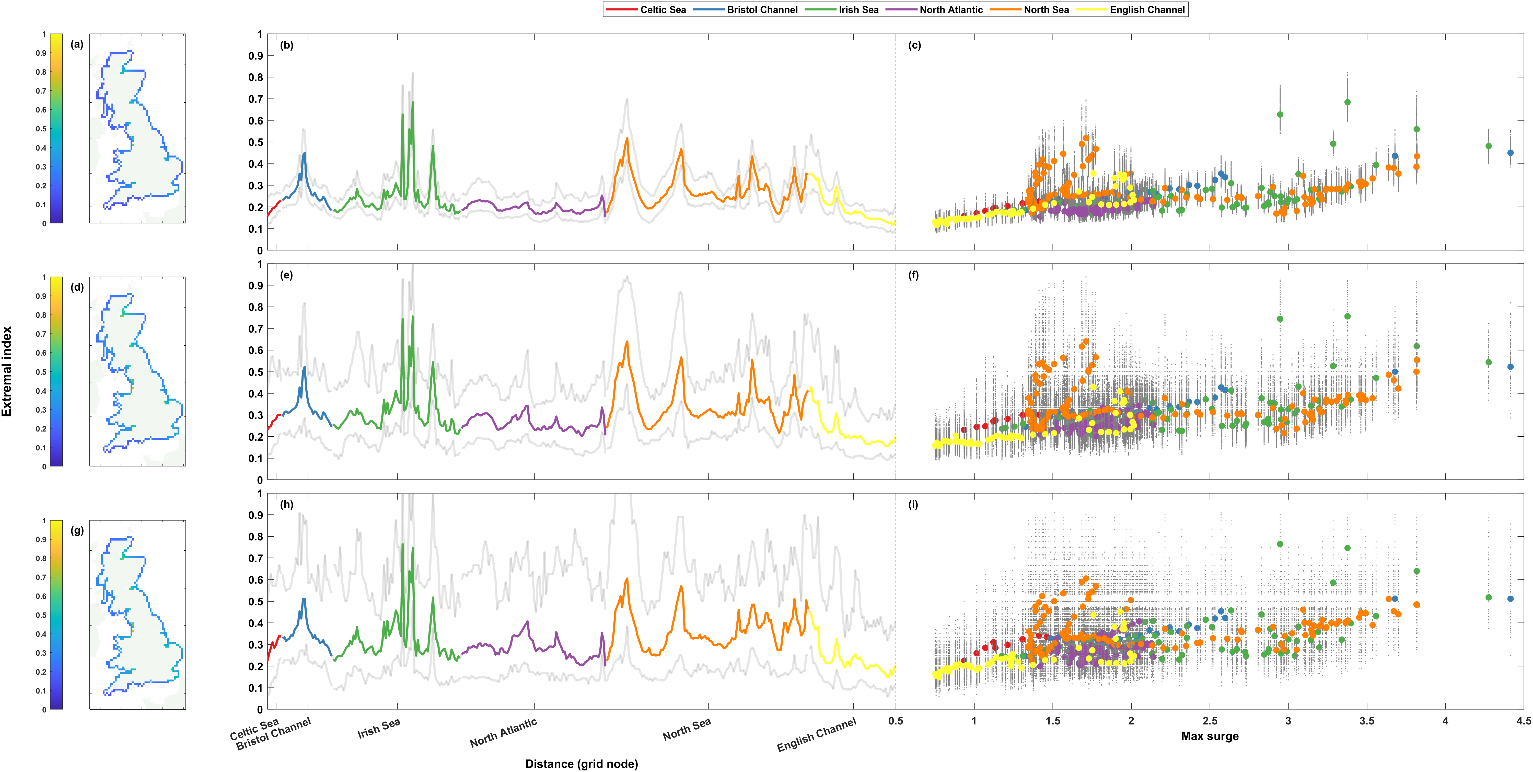


**Fig. S1:** Extremal index (K-gaps) for non-tidal residual timeseries. The top row (**a**-**c**) shows results at the 1 in 1-year return level, the middle row (**d**-**f**) the 1 in 5-year return level, and the bottom row (**g**-**i**) the 1 in 10-year return level. Percentages are shown spatially in panels **a**, **d**, and **g** (left column), clockwise around the coast from the Southwest tip of England ending back at Newlyn in panels **b**, **e**, and **h** (middle column), and finally shown as a scatter plot against maximum non-tidal residual at that grid node in panels **c**, **f**, and **i** (right column). The grey lines (dots) in panels **b**, **e**, and **h** (**c**, **f**, and **i**) show the range of (all) results from the 50-year rolling window analysis.


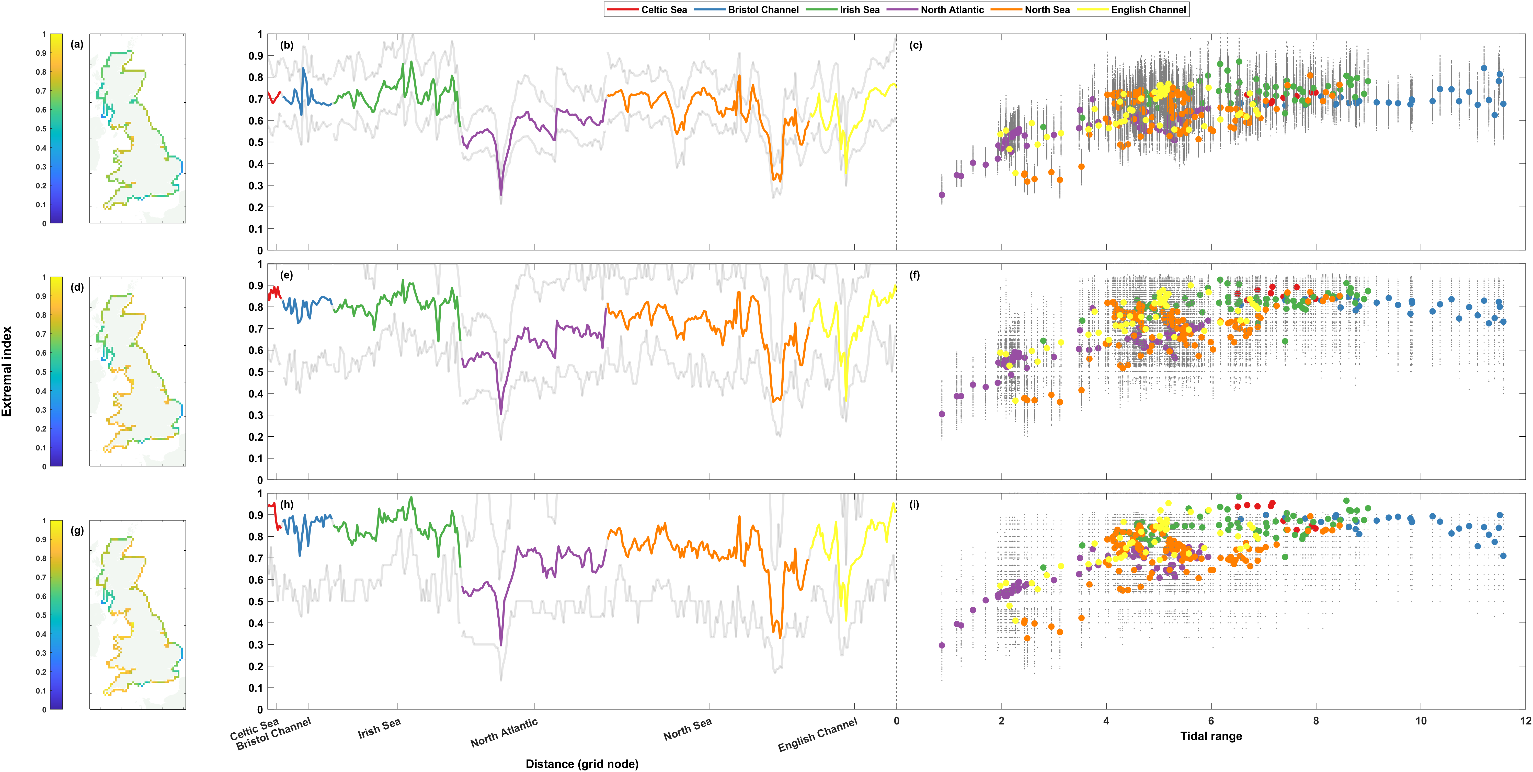


**Fig. S2:** Extremal index (K-gaps) for sea level timeseries. The top row (**a**-**c**) shows results at the 1 in 1-year return level, the middle row (**d**-**f**) the 1 in 5-year return level, and the bottom row (**g**-**i**) the 1 in 10-year return level. Percentages are shown spatially in panels **a**, **d**, and **g** (left column), clockwise around the coast from the Southwest tip of England ending back at Newlyn in panels **b**, **e**, and **h** (middle column), and finally shown as a scatter plot against tidal range at that grid node in panels **c**, **f**, and **i** (right column). The grey lines (dots) in panels **b**, **e**, and **h** (**c**, **f**, and **i**) show the range of (all) results from the 50-year rolling window analysis.
